# Cells sorted off hiPSC-derived kidney organoids coupled with immortalized cells reliably model the proximal tubule

**DOI:** 10.1038/s42003-023-04862-7

**Published:** 2023-05-04

**Authors:** Ramin Banan Sadeghian, Ryohei Ueno, Yuji Takata, Akihiko Kawakami, Cheng Ma, Toshikazu Araoka, Minoru Takasato, Ryuji Yokokawa

**Affiliations:** 1 Department of Micro Engineering, Kyoto University, Kyoto, 615-8540 Japan; 2 Center for iPS Cell Research and Application (CiRA), Kyoto University, Kyoto, 606-8507 Japan; 3 RIKEN Center for Biosystems Dynamics Research (BDR), Kobe, 650-0047 Japan; 4 Graduate School of Medicine, Osaka University, Osaka, 565-0871 Japan; 5 Graduate School of Biostudies, Kyoto University, Kyoto, 606-8501 Japan

**Keywords:** Lab-on-a-chip, Stem-cell biotechnology

## Abstract

Of late, numerous microphysiological systems have been employed to model the renal proximal tubule. Yet there is lack of research on refining the functions of the proximal tubule epithelial layer—selective filtration and reabsorption. In this report, pseudo proximal tubule cells extracted from human-induced pluripotent stem cell-derived kidney organoids are combined and cultured with immortalized proximal tubule cells. It is shown that the cocultured tissue is an impervious epithelium that offers improved levels of certain transporters, extracellular matrix proteins collagen and laminin, and superior glucose transport and P-glycoprotein activity. mRNA expression levels higher than those obtained from each cell type were detected, suggesting an anomalous synergistic crosstalk between the two. Alongside, the improvements in morphological characteristics and performance of the immortalized proximal tubule tissue layer exposed, upon maturation, to human umbilical vein endothelial cells are thoroughly quantified and compared. Glucose and albumin reabsorption, as well as xenobiotic efflux rates through P-glycoprotein were all improved. The data presented abreast highlight the advantages of the cocultured epithelial layer and the non-iPSC-based bilayer. The in vitro models presented herein can be helpful in personalized nephrotoxicity studies.

## Abstract (web summary)

A microfluidic device can help analyse the functions of pseudo proximal tubule cells coming from hiPSC-derived kidney organoids, with the future potential for applications in personalized nephrotoxicity studies.

## Introduction

The proximal tubule located at the outer stripe of the renal medulla is the major site for reabsorption of sodium, water, amino acids, and other essential nutrients such as glucose and albumin that would have been otherwise wasted from the glomerular filtrate^1,2^. The organ is thus crucially important and has been subject to various modeling attempts^3–7^.

Even though recent data clearly highlight the effects of endothelial vasculature on the proximal tubule epithelium^6^, there is still lack of research on optimizing the epithelial tissue itself, as previous models have used either primary cells or immortalized cell lines. One option would be to utilize stem cells for their reproducibility and the ability to offer far better in vivo like characteristics. Nevertheless, there are no sources of stem cells capable of producing nephrons in the adult organ as nephron progenitors are all used up upon birth^8–10^, while obtaining renal progenitor cells from embryonic kidney may seem ethically inappropriate.

Alternatively, such progenitors can be recreated from human-induced pluripotent stem cells (hiPSCs) and differentiated directly into a variety of kidney cell types via stepwise protocols^11–14^. Although recently proximal tubule-like cells have been generated from iPSC lines directly for on-chip assessment purposes^15^, in general, such specialized products lack the 3D niche present in vivo.

To this end, we initiate the idea of extracting cells from hiPSC-derived kidney organoids. hiPSC-derived kidney organoids recapitulate late developmental stages, have a complex 3D structure, and contain cells from all kidney lineages, making them suitable sources of cells with more in vivo like characteristics. Moreover, they do not possess the ethical challenges of obtaining and differentiating embryonic nephron progenitors.

Several studies have put forward the idea of generating kidney organoids by differentiating human pluripotent cells (hPSCs)^13,14,16–18^. However, from the four progenitor populations involved in human kidney development, i.e. nephron, ureteric epithelial, endothelial, and renal interstitial progenitors, most of these protocols create the first two, forming only nephrons or collecting ducts. The differentiation process of hPSCs into kidney organoids introduced by Takasato et al. recapitulates the entire course of human kidney organogenesis^19,20^. It has the advantage of simultaneously producing all the four progenitor populations involved in the formation of kidney organoids over earlier reported methods. In addition, these organoids contain the major structures present in the human kidney. In the context of assessing cellular function it is argued that exposing the organoids to compounds of interest and evaluating the cellular uptake would be problematic because the correct apical/basolateral polarity of the cells cannot be asserted in the aggregate^15^. But what if those cells are extracted and deposited on a 2D platform in a fashion that correct polarity is formed?

While methods for direct differentiation of human embryonic stem cells (hESCs)^21^, hPSCs, and hiPSCs^22^ into proximal tubule-like cells have been reported, and a few applications have been established, the general consensus is that lack of in vivo like conditions such as ECM is the reason for their non-optimal performance^23^.

To tackle this issue a few studies have put forward the idea of mixing these cells with other cell types such as primary cells. For example, recently Beauchamp et al. showed coculturing cardiac fibroblasts with hiPSC cardiomyocytes (hiPSC-CM) prolongs spontaneous beating activity and improves the contraction frequency and amplitude over pure iPSC-CMs^24^. Consequent loss of α-SMA expression indicated a more cardiac tissue-like pattern in the coculture. Of course there are other widely used approaches such as electrical stimulation that may not be applicable to kidney cells^25^.

Here the proximal tubule epithelium was formed on a microphysiological system, referred to as the proximal tubule on a chip (PToC), using cells extracted from hiPSC-derived kidney organoids. Cells that were separated through immunomagnetic cell separation (MACS) were of proximal tubule origin as evidenced by immunostaining against proximal tubule-specific proteins and their gene expression. However, upon cultivation, the expression levels of these markers drop significantly both at mRNA and protein levels, leaving open the question of the organoid-derived cells’ potential to function appositely. In addition, sorted cells tended to retrieve their organoid niche and began to form aggregates, making it impossible to assess the filtration performance of the tissue on the microfluidic chip membrane. We established that such aggregation could be greatly reduced in a 1:1 ratio coculture with immortalized proximal tubule cells. As far as we know, the concept of coculturing two similar cell types, one immortalized and the other obtained from hiPSC-derived organoids has not been investigated yet.

To date, a good deal of both 2D microfluidic and 3D bioprinted models have been developed for drug screening and to mimic the filtration function of the proximal tubule^3–7^. The argument has been that 3D models are superior because they are embedded within an extracellular matrix, and hence can recapitulate the in vivo niche. However, to our knowledge no quantitative analysis has been made to manifest such advantage. In this project, we intend to revisit the conventional view by forming a 2D tissue bilayer with an epithelial/endothelial interspace narrower than that of the 3D bioprinted models reported earlier^7^. Ultimately, we assess the throughput of albumin and glucose reabsorption and cytopempsis processes along with the functionality of the efflux transporter P-glycoprotein (Pgp), demonstrating that even a brief contact with HUVECs substantially improves the filtration rates.

## Results

### hiPSC-derived kidney organoids source pseudo-proximal tubule cells

**Fig. 1:**
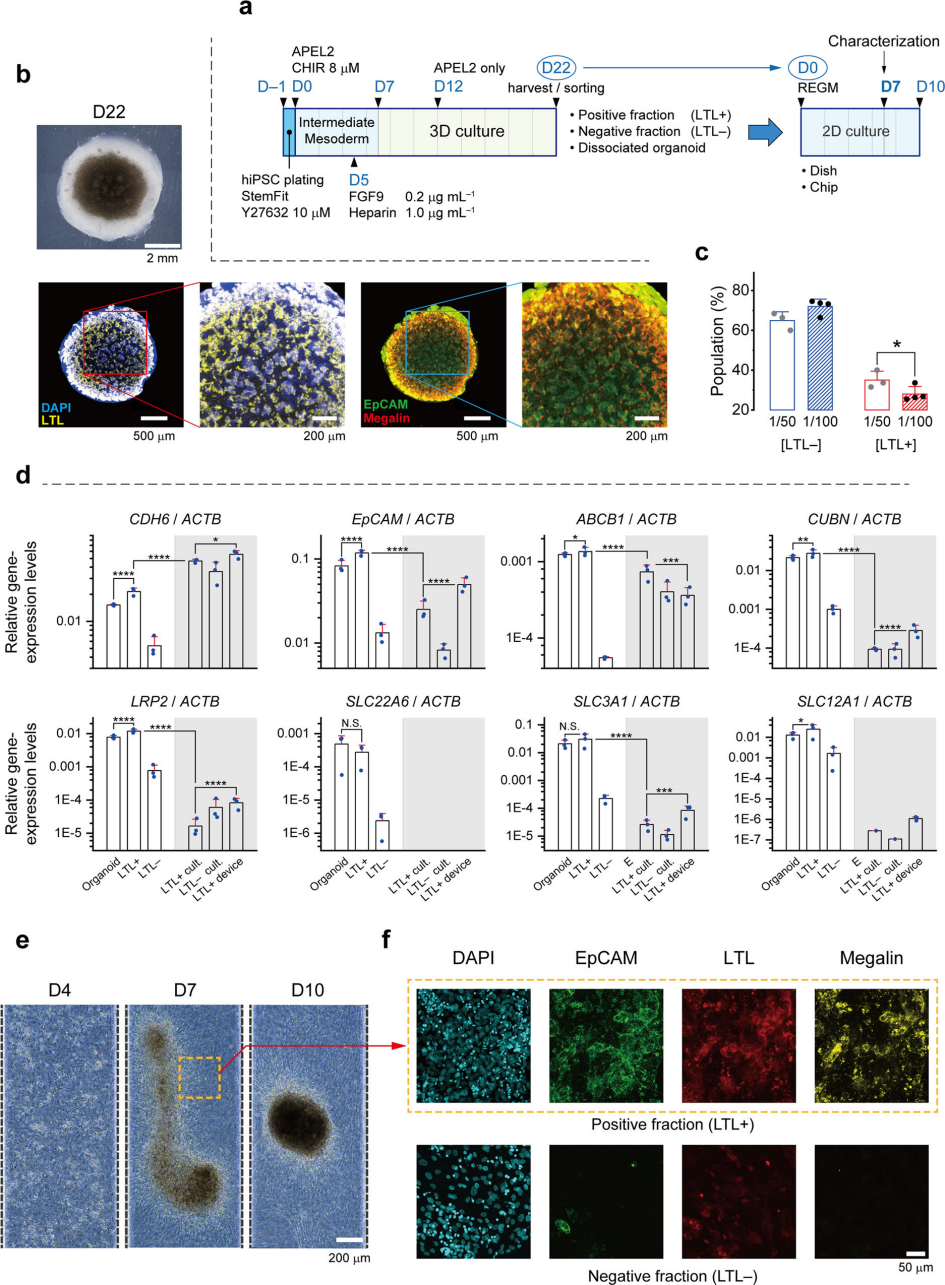
hiPSC-derived kidney organoids source pseudo proximal tubule cells. **a** The simplified protocol of hiPSC differentiation into intermediate mesoderm and formation of 3D kidney organoids, followed by dissociation, sorting, and seeding the cells as sorted. Dissociated organoid, LTL + and LTL–, refer to dissociated organoid cells, positive fraction of MACS, and negative fraction of MACS, respectively. **b** Select brightfield and confocal fluorescent images (z-intensity projected) of kidney organoids on day 22. Immunochemistry is for EpCAM and proximal tubule markers, LTL and megalin. **c** Effect of LTL concentration (dilution factors of 1/50 or 1/100) on the specificity of MACS, *N* = 4 experiments and the error bars represent standard deviation of data, *p < 0.05. **d** Relative gene expression levels obtained from cells as sorted and cultured for 7 days in culture dishes and PToC: Organoid, dissociated organoid cells on day 22; LTL+/–, positive/negative fraction of MACS products. White area corresponds to samples obtained from as-dissociated (organoid) and as-sorted cells, whereas the grayed area indicates samples from cells cultured for 7 days. Error bars represent the standard deviation with *N* = 3 biologically independent experiments. N.S., not significant for *p* > 0.05; *p ≤ 0.05; ** *p* ≤ 0.01; ***p ≤ 0.001; ****p ≤ 0.0001. **e** Evolution of LTL+ cells cultured on the membrane into aggregates, Scale bar, 200 μm. The yellow frame shows the boundary between the cell aggregate and the monolayer. Scale bar, 200 μm. **f** Immunochemistry on day 7 for EpCAM, LTL, and megalin, markers of proximal tubules, in sorted cells extracted from kidney organoids and seeded on the PToC. For the LTL+ tissue fluorescent scans were conducted on select parts at the proximity of aggregates as framed in (**e**). Scale bar, 50 μm.

3D self-organization and nephrogenesis commences at day 7, after harvesting the progenitor cell mixture and pelleting onto transwell filters (Fig. 1a). It was established that the organoid contains optimum percentage and condition of proximal tubule cells on day 22. EpCAM+ population (renal tubules) was identified as superset of proximal tubule cells labeled by a brush border marker, *Lotus Tetragonolobus Lectin* (LTL), and more specifically by the albumin transporter, megalin (Fig. 1b). According to the earlier estimates, the organoid roughly contains a 20-30% population of proximal tubule cells^19^. A 3D confocal reconstruction of the organoid is presented (Supplementary Movies 1 and 2).

On day 22 the organoids were dissociated into a cell suspension. The suspension was partially exposed to LTL and subjected to MACS after the optimum antibody concentration was determined (Fig. 1c). (Also see ‘Methods’ section and Supplementary Table 1 for details.) We also examined FACS for its higher degree of specificity and characterized the products as-sorted and cultured (Supplementary Fig. 1, 2 and Supplementary Data 1). However, eventually we proceeded with MACS because of its capability to produce cells with phenotypic features similar enough to primary proximal tubule cells (Supplementary Movies 3–6). Subsequently, the positive and negative fractions of the MACS products were collected and cultured for 7 days in 2D conditions or lysed, as-sorted, along with rest of the cell suspension. To determine if the proximal tubule compartment of the nephron was present in our organoids and examine the effect of 2D culture, we conducted mRNA analysis against a number of relevant genes.

We found that the expression level of *CDH6* (K-cadherin), a specific marker of the proximal tubule progenitor cells^16,26,27^, increased during culture while the increase was more pronounced for the negative fraction (LTL–). Namely, the increases were 2.2-fold and 6.2-fold for the LTL+ and LTL– populations, respectively (Fig. 1d). In contrast, the 2D environment was not favorable for the rest of markers examined, as the expression levels dropped (*ABCB1*, *CUBN*, *LRP2*, *SLC3A1*, and possibly *SLC12A1*), or totally diminished (*SLC22A6* (OAT1)).

The tissue cultivated from LTL+ cells cultured in the chips (Supplementary Fig. 3a,b) began to aggregate and form tiny spheroid-like agglomerates on the membrane (Fig. 1e). Nevertheless, immunostaining confirmed the abundance of proximal tubule proteins in these cells. EpCAM, LTL, and megalin appeared with high contrast, solely in LTL+ cell aggregates that were formed slowly and became discernable around D7 (Fig. 1f). No aggregates were observed in the LTL– tissue layer (Supplementary Fig. 3c).

### Introducing the LTL+/RPTEC coculture system

**Fig. 2:**
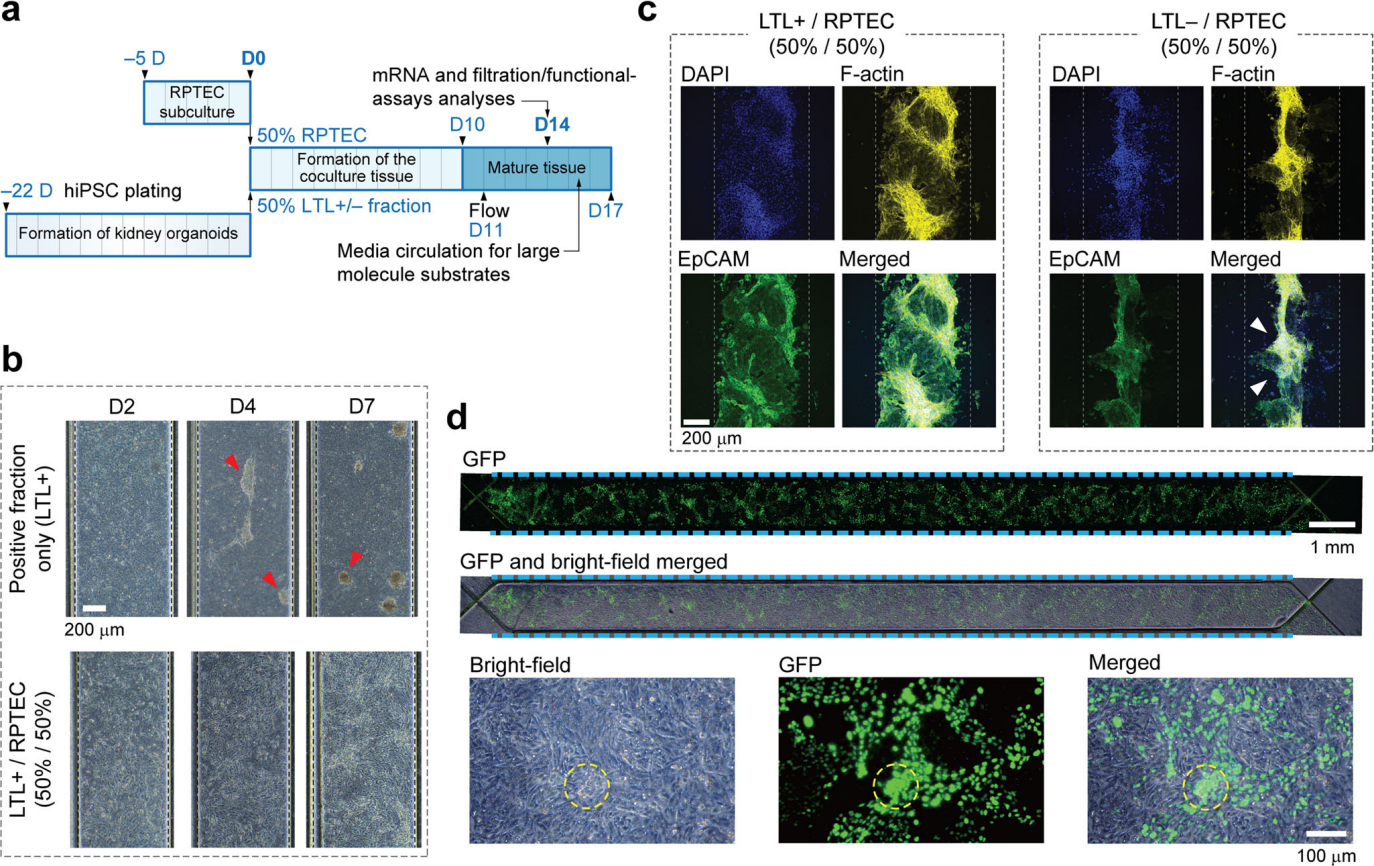
Coculture of kidney organoid MACS products with RPTECs. **a** Timeline (in days) demonstrating the coculture assay. hiPSCs are plated 23 days prior to the coculture as outlined earlier, followed by RPTECs which are subcultured on day –5. Kidney organoids are harvested upon maturation, dissociated, and mixed in equal portions with RPTECs. The cell mixture is then introduced into the chip. **b** Brightfield images showing the evolution of LTL+ and cocultured tissues with time. The positive fraction-only cells begin to aggregate from ∼D4 and form separable spheroids by D7 (as indicated by red arrowheads), rendering the device impractical for filtration assessments. Whereas no detachment is observed in the coculture with RPTECs. Scale bar, 200 μm. **c** Z-intensity projected fluorescent images taken from immunostained samples on D7. DAPI, F-actin and EpCAM are represented in blue, yellow, and green, respectively. Once cocultured with RPTECs at a 50/50 ratio, LTL+ cells blend well and make a confluent tissue layer. Coculturing the LTL– fraction with RPTECs however, does not yield a confluent tissue layer. The tissue partially detaches as indicated by white arrowheads. EpCAM is faintly expressed throughout this tissue and mostly in the RPTECs. Gray dashed lines show the whereabouts of the PToC membrane. Scale bar, 200 μm. **d** Fluorescent, brightfield and merged images of the cocultured tissue on D14. Top rows show that the channel is covered by RPTECs and LTL+ cells equally in its entirety. High magnification pictures at the bottom row elucidate that the dissociated/sorted cells in the mixture not only form small aggregates but are also distributed almost evenly throughout the coculture tissue. Scale bars are in 1 mm and 100 μm on top and bottom rows, respectively. Dashed lines are to guide the eye.

Following MACS, both LTL+ and LTL– fractions were collected and cultured at 10^7^ cells mL^–1^. In addition, a 50:50 coculture of each of the fractions with immortalized renal proximal tubule epithelial cells (RPTEC/TERT1 cells, hereafter referred to as RPTECs) were examined (Fig. 2a). As shown in the bright field images (Fig. 2b), LTL+ cells retrieved their organoid niche and began to aggregate. However, once cocultured with RPTEC cells they flattened out, making it possible to study the barrier function. It became clear that LTL+ and RPTEC cells blended well, provided a confluent layer, and were positive to EpCAM (Fig. 2c). Whereas in contrast, although LTL– cells in the mixture were clustered they could not coalesce into a continuous tissue. The LTL+/RPTEC epithelial layer was hereafter referred to as the coculture. Confocal laser scans directed from the apical to the basal sides show the expression of apical (LTL, ZO-1, and Pgp) and basolateral (EpCAM) proximal tubule markers (Supplementary Movies 7–9).

For preliminary trials GFP-positive hiPSCs were employed in cultivating the organoid in order to ease tracking of the sorted cells. It became clear that in the coculture tissue both cell types maintain their conformality over the entire layer. Organoid-derived cells that populate ∼50% of the epithelium are discernable (Fig. 2d). By day 14 the aggregates were assimilated into the coculture, although some clusters still remained. At this stage the tissue was deemed ready to be characterized.

### RPTEC and LTL+ synergy, improved mRNA levels and filtration capacity

**Fig. 3:**
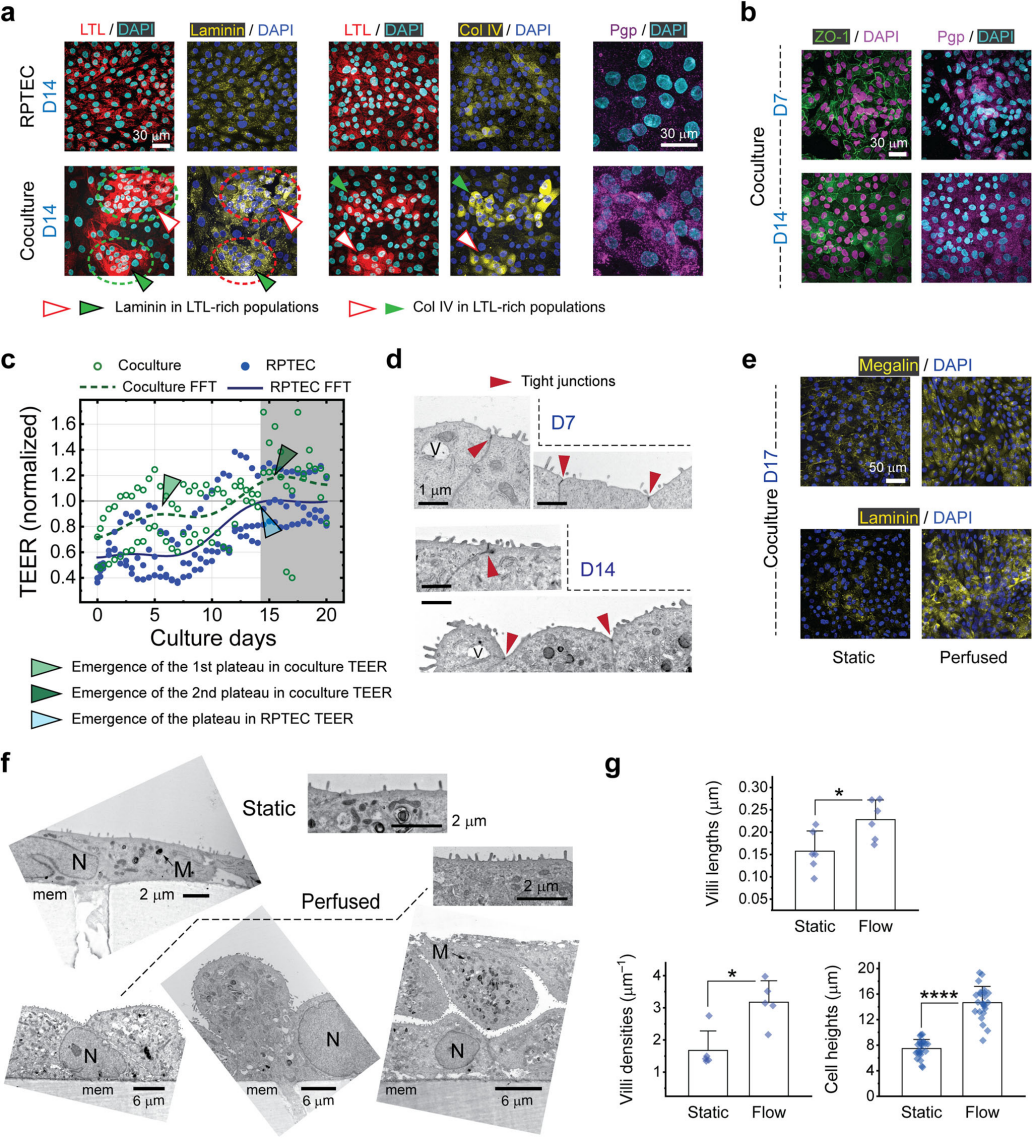
Comprehensive evaluation of the coculture tissue. **a** Immunohistochemistry for LTL, laminin-111 (α1β1γ1) heterotrimer, collagen IV, and Pgp in single layer RPTEC and cocultured tissue layers on day 14, highlighting the enhancement of both ECM proteins in the latter. Laminin- and collagen-bound clusters, morphologically resembling islet-epithelial clusters in vivo, appear mostly around LTL+ cells sorted off the organoids, as indicated by white and green arrowheads. Same applies for the proximal tubule apical protein, Pgp. Scale bars, 30 μm. **b** Appearance of tight junctions around LTL+ cell clusters on D7 and throughout the entire coculture tissue on D14. In (**a**) and (**b**), picture pares are taken from the same sample and scale bars are 30 μm. **c** Evolution of the transepithelial electric resistance (TEER) with culture time for the RPTEC-only and coculture tissue layers. In the coculture system the resistance increases more steeply and reaches the first plateau of two (light green arrowhead), possibly on account of smaller LTL+ cells in the mixture. The second plateau (dark green arrowhead) happens at almost the same time the resistance saturates in the RPTEC-only system, and it corresponds to the contribution of RPTECs in the mixture (light blue arrowhead). TEER is normalized to the final steady-state value of the RPTEC-only tissue. *N* = 3 and 2 independent experiments for RPTEC-only and coculture cases, respectively. **d** TEM micrographs highlighting emergence and establishment of tight junctions (dark red arrowheads) in the coculture monolayers on D7 and D14. Scale bar, 1 μm. V, vacuole. **e** Immunocytochemistry for megalin and laminin-111 shows improvement in expression intensity and distribution of both proteins in the coculture caused by flow-induced shear stress. Scale bar, 50 μm (**f**) TEM micrographs of representative cells from the coculture system compare static culture and perfusion culture conditions. Formation of various epithelial tissue morphologies such as flat monolayers (that cover almost the entire area of the membrane), monolayers with cell protrusions, and front to front (apical-apical) cell stacks is revealed in the latter. All the TEM micrographs are from the cells fixated on D14. Scale bars represent 2 μm and 6 μm for micrographs representing static (top) and perfused (bottom) conditions, respectively. M, mitochondria; N, nucleus; mem, PET membrane. **g** Quantification of the microvilli length/density and cell height in the coculture system on D14. We define the villi density as the count of protrusions divided by the length of the cell membrane cross-section periphery, measured from individual snapshots. Flow induced shear stress manifested growth of not only longer and denser microvilli but also taller cells evidencing for a squamous to cuboidal morphology translation. All samples are on D14. For quantification purposes, from a total of *N* = 3 independent TEM experiments per condition, *n* = 6, 5, and 24 cells were randomly selected to measure villi lengths, villi densities, and cell heights, respectively. Error bars represent the standard deviation of data.

Apparently, the brush border marker is expressed more strongly in kidney organoid-derived LTL+ cells than in the RPTECs. Also, we observed that in the coculture tissue LTL+ cells secrete their own ECM in the proximity of RPTECs since both laminin and collagen IV appear densely where LTL antibody is strongly expressed. Intense expression of Pgp in the coculture may suggest an improved capacity in the elimination of exogenous substances. (Fig. 3a).

The synergistic effect of combining LTL+ cells with RPTECs is also evidenced by strong appearance of the tight junction protein. ZO-1 appears early on D7 in the coculture (Fig. 3b), corresponding with the first plateau observed in the TEER profile of Fig. 3c. In these plots solid and dashed lines represent smoothed estimation of the TEER trends for the RPTEC and coculture cases, respectively. The first plateau occurs at an earlier date in the coculture (as shown by a light green arrowhead), indicating a shorter contact inhibition period. LTL+ cells are smaller in average and exhibit a steeper log phase. The resistances converge around day 14, with the coculture (dark green arrowhead) being slightly more resistive that the RPTEC tissue (light blue arrowhead). Tight junctions are visualized in the TEM images as well (Fig. 3d). Our TEER measurement strategy and setup have been reported earlier^28,29^. Bright filed images show the evolution of tissue culture layers on the TEER chips with time (Supplementary Fig. 3d). Clear barrier function of the epithelial layer was verified prior to conducting transport measurements on D14 (Supplementary Fig. 3e).

To demonstrate and quantify possible effects of mechanical stimulus we exposed the apical side of the tissue to flow-induced shear stress. The effect of media flow on the tissue culture is twofold. As opposed to stagnant condition, flow not only helps to replenish crucial components of the media, e.g. growth factors and hormones but also exerts shear stress on the underlying tissue. These two effects have been rarely distinguished in the literature of renal microphysiological systems. Here we circulated the culture media at the static culture condition as well, but with a miniscule flow rate (1 μL min^–1^) to ensure that the shear stress would be negligible while maintaining the supply of media components. In this manner we could distinctly discern and study the effect of mechanical stimulus on the apical side of the endothelium. Proximal tubule cells are known to respond to shear stimulus within a certain range. The continuous shear stress along the proximal tubule is measured to vary from 0.1 to more than 1 dyne cm^–2^ in vivo^30,31^. However, due to the tubuloglomerular feedback the instantaneous flow into the tubule might be pulsatile rather than smooth^32^. It is expected that at a given rate a pulsatile flow would be more stimulating.

Previously Long et al. observed that cell differentiation, proliferation, and to certain extent endocytosis were enhanced at lower shear stress levels^33^. In the perfused media condition, we circulated the medium at a rate of 10 μL min^–1^ resulting an average shear stress of 0.06 dyne cm^–2^ which is about the minimum reported values in vitro. As a result, we could infer that the instantaneously pulsating nature of the peristaltic pump (Supplementary Fig. 4a, b) may play a role in the improvements in cellular morphology and function observed herein. Immunostaining revealed even such a small amount of shear stress remarkably affects the intensity and distribution of megalin in the mature epithelia of the coculture and non-iPSC bilayer systems.

Various morphological and functional factors were improved. Megalin, a cotransporter of albumin, was redistributed in the cytosol and it is evident that laminin secretion has been improved (Fig. 3e). Electron microscopy also revealed significant alterations in morphology of cells. Aside from the characteristic features of mature proximal tubule cells with brush border structures, vacuole- and mitochondria-rich areas were observed in the cocultured cells. Cells exposed to flowing medium for only 3 days underwent apical and basal surface reduction that is indicative of squamous to cuboidal phenotype change (Fig. 3f)^34,35^. We were also able to quantify some of these geometrical changes by analyzing a large number of TEM images. As illustrated in the graphs of Fig. 3g, cocultured cells grew taller and developed longer and denser microvilli. For such quantification purposes we solely counted the cells in the monolayer and disregarded the small fractions of the tissue containing stratified cells.

**Fig. 4:**
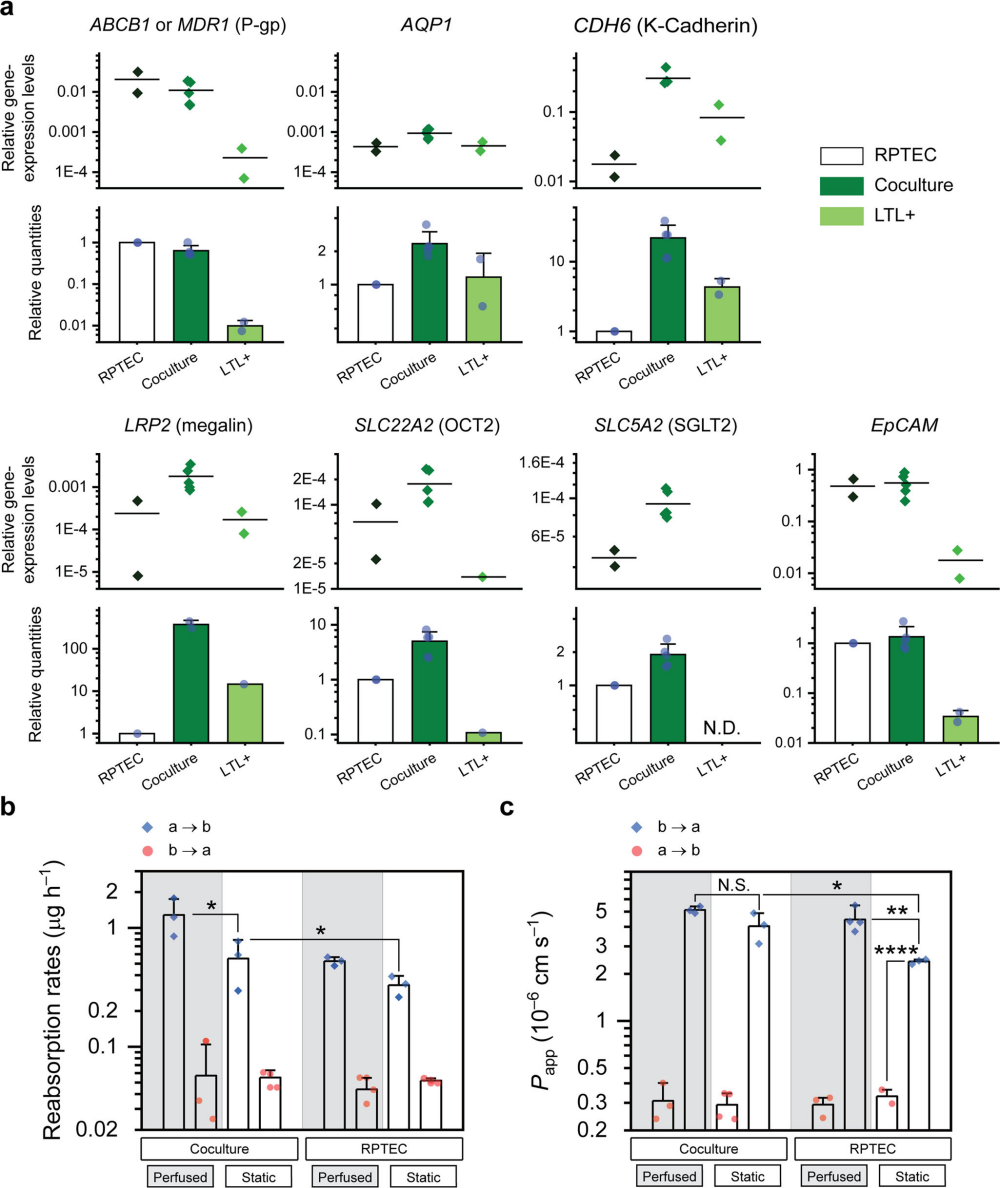
Improved mRNA expression in the coculture followed by improved filtration capacity. **a** Expression levels of certain genes in the RPTEC/LTL+ coculture show improvement over those of each component. Functional, *ABCB1* (*MDR1*), *AQP1*, *LRP2*, *SLC22A2* (OCT2), *SLC5A2* (SGLT2), and structural, *CDH6* (K-Cadherin) and *EpCAM*, relative gene expression levels (versus those of the *ACTB* level), are accompanied by relative quantities (normalized to the values of the RPTEC tissue). Except for *ABCB1* and EpCAM, the expression levels of other genes were significantly increased in the cocultured tissue. There was no detrimental effect on mRNA expression levels; *N* = 2 and 5 biologically independent experiments for RPTEC/LTL+ monocultures and the coculture case, respectively; Error bars represent the standard deviation of data. Reabsorption rates of glucose (**b**) and apparent permeabilities (*P*_app_) to Rh123 (**c**) measured on D14 for both RPTEC-only and cocultured tissue layers subjected to static and perfused culture conditions. Vectorial transport is verified in all cases. Transfer rates were estimated by conducting linear regression on time course data. a → b, apical to basal; b → a, basal to apical; Measurements taken from a minimum of *N* = 3 independent chips; Error bars represent SD of the mean; N.S., not significant for *p* > 0.05; *p ≤ 0.05; **p ≤ 0.01; ***p ≤ 0.001; ****p ≤ 0.0001.

Compared to either cell source, the mRNA expression levels of certain key genes were elevated in the cocultured epithelium (Fig. 4a). However, discrepancies were observed between these levels and the protein expression levels of the corresponding markers, as revealed by immunostaining. For example, although ABCB1 was expressed at almost the same level in the RPTEC and coculture tissue samples, Pgp was more abundantly found in the latter. The glucose and albumin transporters, *SGLT2*, and *LRP2*, respectively, were both elevated in mRNA and protein levels. *LRP2* was surprisingly increased more than 100-fold.

We hypothesized such increase in the transporter protein levels could lead to improved active transepithelial transport. To test our hypothesis, we quantified the reabsorption rate of glucose and the excretion rate of rhodamine 123 (Rh123), as measures of *SGLT2* and Pgp activity, respectively. We showed that the apical to basal (a → b) transport rates of glucose in the coculture were improved by about 1.75 and 2.50 times over that of the RPTEC-only monolayer in static and perfused culture conditions, respectively (Fig. 4b). Among all four conditions examined, while a → b transfer rates of the substrate were significantly higher than those of the reverse (b → a) direction, there were no significant changes in the reverse transfer rates. Both observations confirm the vectorial nature of glucose transport.

Apparent permeabilities (*P*_app_) to the Pgp substrate, Rh123, are shown in Fig. 4c. Since the excretion rates (b → a) are higher than the absorption rates (a → b), the transport is unidirectional in all four cases examined. In addition, the efflux ratio of the substrate, defined as the ratio of excretion to absorption rates, has been increased by 90% in the coculture system under static culture condition, confirming substantial improvement in xenobiotic deposition capacity.

### Establishing and characterizing the RPTEC/HUVEC bilayer chip alongside

**Fig. 5:**
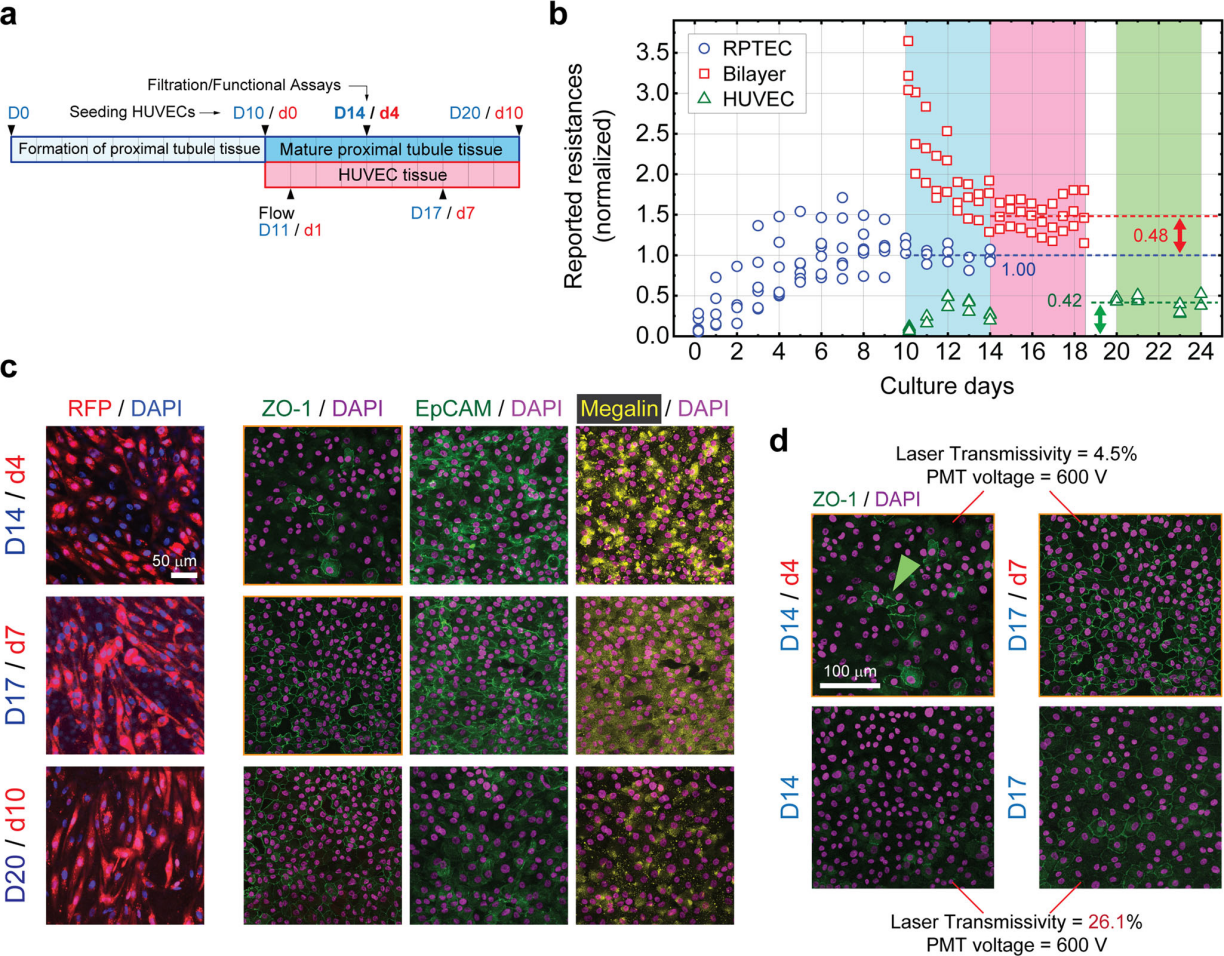
Establishment of the non-iPSC bilayer system. **a** The timetable of generic cell seeding and maintenance processes. HUVECs are not included for the single-layer devices. **b** Time course of reported resistances of the RPTEC-only (blue circles), bilayer (red squares), and HUVEC-only (green triangles) tissue layers. For the bilayer devices once HUVECs are introduced on D10, the overall resistance jumps upon formation of a confluent endothelial layer and then starts to decline until reaching steady state on D14/d4. Dashed lines indicate the average resistances obtained over the last 4 days of culture (colored bars) for each case. All the measured values are normalized to that of the RPTEC TEER at steady state, i.e. 60 Ω cm^2^ (blue dashed line). Green and red two-headed arrows indicate, respectively, the reported resistance of the HUVEC-only layer and the resistance increment measured upon addition of HUVECs to the RPTEC-only tissue. A stable bilayer is formed on D14/d4. RPTECs were cultured on *N* = 6 devices, out of which *N* = 3 were rendered bilayer upon addition of HUVECs on D10. *N* = 3 devices were dedicated for the HUVEC-only tissue. **c** Immunohistochemistry for ZO-1, EpCAM, and megalin in RPTEC tissue of the bilayer system along with fluorescent images of corresponding RFP-tagged HUVECs, showing the evolution of tight junctions, degree of re-epithelialization, and distribution of the albumin transporter, respectively. Tissue activity and integrity is peaked between D14/d4 and D17/d7. Scale bar, 50 μm. **d** Effect of HUVECs on the intensity of ZO-1 expression; In the presence of HUVECs tight junctions appeared from D14/d4 (green arrowhead) onward and became clearly visible on D17/d7, while in the absence of HUVECs they appeared faintly, even at a considerably higher confocal laser transmissivity. No Triton X was used in this experiment. Scale bars are 100 μm. All fluorescent images are confocal z-intensity projected. Scale bar, 100 μm.

To create completely isolated proximal tubule epithelial and peritubular endothelial channels we adapted our protocol to ensure a thoroughly sealed RPTEC tissue is formed before introduction of HUVECs (Fig. 5a). This not only prevented intermix of EGM2 and REGM that would have otherwise caused detachment of HUVECs (Supplementary Fig. 4c, d) but also allowed us to study the effects of endothelial cells on a maturated proximal tubule epithelial tissue. Prior to seeding we coated the membrane at both sides solely with a cell adhesion agent and allowed the cells to secrete their own ECM. While no external ECM protein was applied, the bilayer was safely maintained for at least 10 days after which HUVECs began to detach (Supplementary Fig. 4e and Supplementary Movies 10 and 11).

Similar to the single-layer coculture system, we probed the overall electrical resistance of the RPTEC/HUVEC system during the entire course of culture in intervals. Devices with single-layer RPTEC and HUVEC tissue were used for comparison and all values were scaled with respect to the average RPTEC TEER measured during last 4 days of culture, that is 60 Ω cm^2^ (Fig. 5b). The overall resistance overshoots during formation of a confluent HUVEC monolayer from D10/d0 to D13/d3 and then plateaus off past d4. However, the amplitude of resistance jump upon creation of the bilayer, as indicated by the red arrow in Fig. 5b, is not significantly different from the resistance of the HUVEC-only tissue (green arrow). As a result, even though the bilayer resistance profile seems stable during the culture, it is unlikely that introducing HUVECs has significantly affected the transepithelial resistance of the RPTEC layer. Usually, RPTEC monolayer cultures exhibit TEERs around 0.1 to 1 kΩ cm^2 ^^36,37^, while the proximal tubule tissue in vivo is known to display values that are an order of magnitude smaller. It is often argued that the leakier barrier function of the tissue in vivo is due to a higher transcellular or paracellular transport^38^. Apparently, and possibly for the same reason, the resistance values of a few ten Ω cm^2^ measured herein lie between those of the previously reported values for RPTEC monolayers and the proximal tubule tissue in vivo. Later we establish that HUVECs indeed play a role in such improvement.

It is well established that a paracrine signaling network is formed once RPTECs are cocultured with endothelial cells, leading to improved RPTEC proliferation rate and differentiation^39^. In our case, evidence of interaction and such signaling between HUVECs and RPTECs was discernable from d4 of the bilayer formation. We observed that in the presence of HUVECs, epithelial tissue integrity and possibly function both peak sometime between days 4 and 7 of the bilayer formation as evidenced by unwavering appearance of EpCAM/ZO-1, and expression of maximally dispersed (evenly distributed) megalin on d7 (Fig. 5c). Since megalin is a mobile albumin transporter an improved cytosolic delivery of albumin, already present in culture medium, is concluded.

This is noticeable because in contrast to some earlier reports where both cell types were brought together at almost the same time^6^, we observe improvement of proximal tubule tissue integrity and function even after 10 d of culture. As observed by cross-sectional transmission electron microscopy (TEM), 3 μm-wide pores of the PET membrane were the only possible channels through which these two cell types could communicate (Supplementary Fig. 3b). Indeed, a porosity of ∼5% was enough to establish sufficient cell-cell contact. In addition, the expression level of RPTEC tight junction protein has been distinctly increased in the bilayer system (Fig. 5d) similar to an earlier report^6^, and so is its appearance frequency. As a result, all the characterizations and functional assessments were centered on D14 of the epithelial layer culture regardless of the cell source. Notice that ZO-1 was expressed vividly in the coculture tissue on D14, whereas it has faintly appeared in the RPTEC-only single layer system at the same culture condition. The RPTEC/HUVEC bilayer system was subjected to a similar degree of scrutiny as the coculture tissue.

### Protein profiling clarify morphological enhancements in proximal tubule cells

**Fig. 6:**
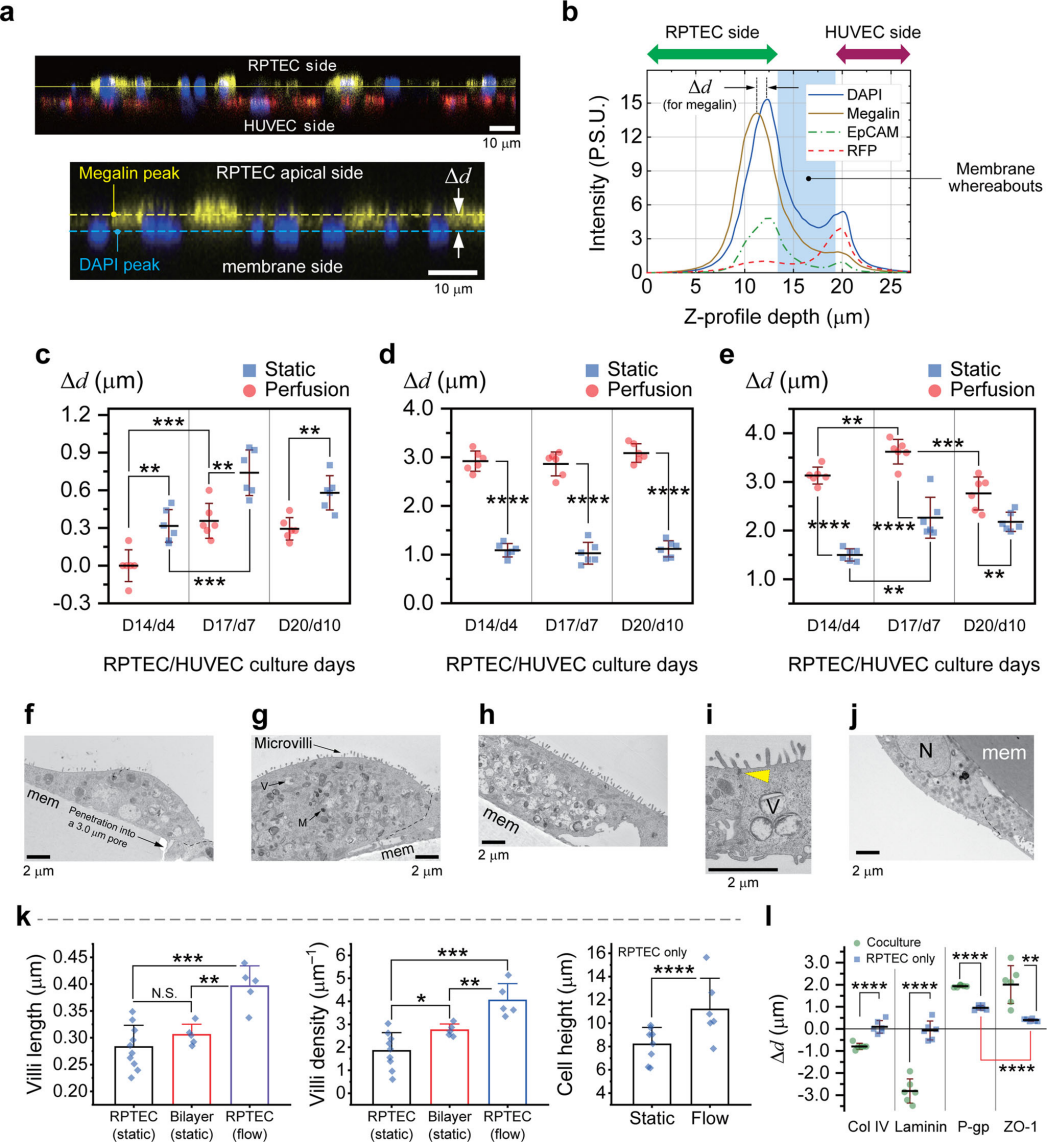
Characterization of the RPTEC layer in the bilayer system using z-intensity profiling of proteins and TEM to show the effect of flow. **a** Select cross sectional fluorescent confocal image of the RPTEC/HUVEC bilayer system, immunostained for megalin, illustrating the definition of the relative distance of the protein of interest (e.g. megalin) measured from the center of nuclei. Scale bars are 10 μm. **b** Representative z-intensity profiles of fluorescent signals obtained by averaging emission intensities of various markers throughout a laser scan area of 0.1 cm^2^ with a Δ*z* (pitch) of 200 nm. Average proximal tubule-specific marker-to-nuclei distance in RPTECs (bilayer) obtained under static and perfused culture conditions for (**c**) megalin, (**d**) LTL, and (**e**) SGLT2; *N* = 2 independent chips were used to make *n* = 3 random measurements from each; Error bars indicate standard deviation. Statistically significant differences between data pairs are indicated by asterisks, *, **, ***, ****, for *p* ≤ 0.05, 0.01, 0.001, and 0.0001, respectively. Select cross-sectional TEM images highlight the appearance of the apical membrane of RPTECs in various culture conditions on D14/d4: (**f**) single layer under static, (**g**) bilayer under static, and (**h**) single layer under perfusion culture conditions. The close-up TEM of (**i**) reveals a tight junction (yellow arrowhead) formed between two adjacent RPTECs under perfusion culture. **j** A HUVEC at the opposite side of the membrane. Scale bars are 2 μm. All the micrographs are from the cells fixated on D14/d4. M, mitochondria; N, nucleus; V, vacuole; mem, PET membrane, arrows point to tight junctions, and the dashed lines show cell-cell boundaries. **k** Quantification of the microvilli length/density and the RPTEC height. We define the villi density as the count of protrusions divided by the length of the cell membrane cross-section periphery, measured from individual snapshots. RPTECs/HUVECs are on D14/d4. Clearly RPTECs in the proximity of HUVECs developed denser apical microvilli, resulting a higher surface area. In addition, the microvilli density distribution is narrower in the bilayer configuration. No significant difference in villi lengths was observed between the single layer and bilayer cases in static culture condition. Flow induced shear stress manifested not only longer but also a larger density of microvilli. For quantification purposes, from a total of *N* = 3 independent TEM observations per condition, *n* = 9, 5, and 5 RPTEC micrographs were randomly selected from single layer (static), bilayer (static), and single layer (flow) culture conditions, respectively, to measure villi lengths and densities. To measure cell heights, *n* = 8 and *n* = 9 micrographs were examined for static and flow conditions, respectively. Error bars represent the standard deviation of data. **l** Average distances of basement and apical proteins from the nucleus illustrated for RPTEC-only and coculture tissues developed under flow culture condition. Data obtained by z-intensity profiling of fluorescent images. Statistics are derived in a similar fashion to (**c**–**e**).

We estimated the spatiotemporal expression patterns of certain proximal tubule-specific proteins by conducting extensive immunofluorescence observations. Briefly, fluorescent intensities representing the expression levels of several antibodies were quantified by analyzing stacks of confocal images taken across the cell-laden membranes. The procedure is illustrated using representative cross-sectional fluorescent images of the RPTEC/HUVEC bilayer and the corresponding intensity profiles (Fig. 6a, b). Δ*d* denotes the distance between the positions of the peak expression level of each antibody and the peak expression level of DAPI, as a measure of nuclei whereabouts. By plotting values of Δ*d* against culture days we could trace the motility of the proteins in the epithelial layer.

For the case of megalin, the farther it appears from the nuclei the higher chance of it being bound to the apical membrane, i.e., remain at an inactive state. We noticed that in the bilayer system if HUVECs are seeded first, megalin which is initially expressed at the apical membrane, becomes internalized during 7 days of RPTEC culture (Supplementary Fig. 5a). Whereas, when RPTECs are seeded first, it would be completely taken into the intracellular milieu on D14, suggesting a peak endocytosis activity (Fig. 6c and Supplementary Fig. 5b)^40^. Data presented herein and the fluorescent images of Fig. 3e make clear that perfusion not only increases the activity of megalin but also its abundance in the cytosol. Such improvement is reflected in a higher reabsorption rate of albumin. In line with previous reports of cell height/compactness increase under fluid shear stress^31^, by protein profiling here we found that the apical markers, LTL and SGLT2, appear more distant from the nucleus (Fig. 6d, e).

Cross-sectional electron microscopy of the cells on the top layer at day 14 revealed the distinctive effects of shear stress and proximity with HUVECs on the non-iPSC epithelia as well (Fig. 6f–j). Self-secreted ECM components diffused through the membrane pores providing a communication path with HUVECs. Even though the cells were squamous in comparison with 3D tubular structures^5,7^, tight junctions were clearly discernable. Observation of tight junctions confirms the impervious nature of both RPTEC-only and RPTEC/LTL+ coculture epithelia and is essential to validate our transcellular transport assessments. Tight junctions in the RPTEC-only tissue began to form at a later stage than those in the coculture monolayer system. It also appears that they become tighter under perfusion culture, although this has not been statistically confirmed (Supplementary Fig. 6).

Regardless of the epithelial cell source, the density and length of villi as well as the cell height, all increased under the influence of shear stress, resembling the condition in vivo. Interestingly, even in the absence of mechanical stimuli denser villi appeared on RPTECs in contact with HUVECs (Fig. 6k). Larger and denser population of villi manifest in a more efficient endocytosis and transcytosis.

On D14 we measured the average relative distances of basement (laminin & collagen IV), subapical (ZO-1), and apical (Pgp) proteins from the nucleus in the single layer RPTEC-only and coculture systems (Fig. 6l). Since both the ECM and the apical proteins are expressed farther from the nuclei in the cocultured tissue, it is gathered that the proximal tubule cells of the coculture tissue grew taller in average. Undoubtedly, RPTEC tissue has a more uniform thickness across the membrane and the collective degree of cell orientation and polarization is higher (smaller error bars for Pgp and ZO-1). Also notice that ZO-1 is expressed clearly beneath the apical Pgp in the RPTEC-only tissue. Quantification of cell heights based on TEM observations of individual cells did not quite lead to the same results as those presented in Fig. 6l; however, the trend was similar. We believe the discrepancy roots in the process of z-intensity profiling where the average relative distances measured between apical and basal markers are shortened because the PET membrane is not flat. In addition, since apical proteins are not necessarily expressed right on the peak of the cells measuring cell heights from the membrane to the cell apex would result in larger values. Having said that the effect of shear stress on cell height was more pronounced in the coculture, i.e. 37% increase in the RPTEC only tissue versus 99% in the coculture (cell heights in Fig. 3g and Fig. 6k).

### Investigating the influence of shear stress and proximity to HUVECs on RPTEC mRNA levels and filtration performance

**Fig. 7:**
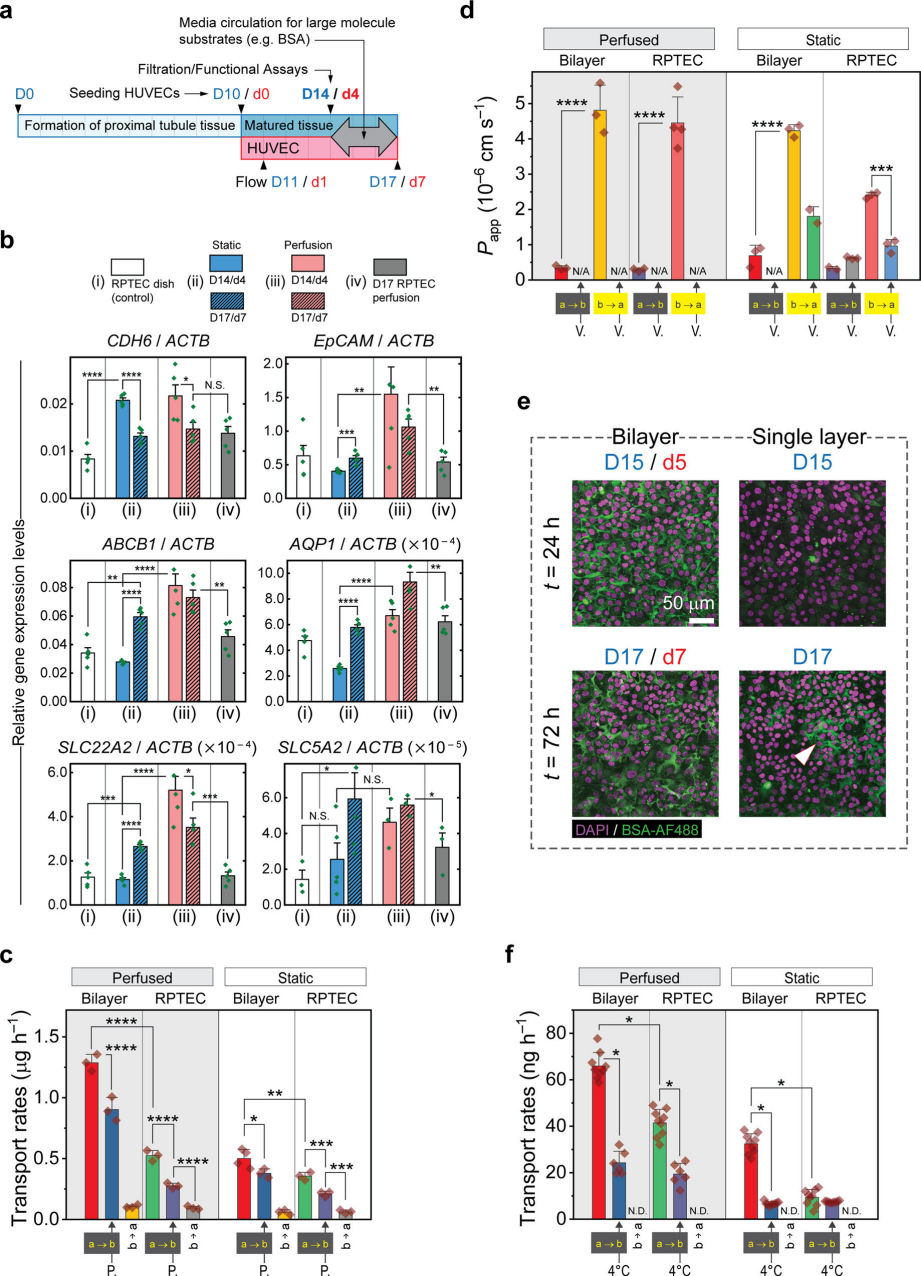
Assessment of the filtration capacity of RPTEC tissue showing the effects of HUVECs and flow induced shear stress. **a** The generic protocol for quantification of renal reabsorption and excretion using the PToC. Measurements commence on day 14 irrespective of the assay in hand and the tissue layer configuration. The media in both epithelial and vasculature microchannels are circulated to improve diffusion of large-molecule substrates (e.g. BSA-AF488). Transfer rates (reabsorption of albumin/glucose or apparent permeability to Rh123) were estimated by conducting linear regression on time course data. **b** Relative expression levels (vs. *ACTB*) of structural genes, *CDH6* (K-Cadherin) and *EpCAM*, and functional genes, namely, *ABCB1* (*MDR1*), *AQP1*, *SLC22A2* (OCT2), *SLC5A2* (SGLT2), vs. the level of *ACTB* gene in the RPTEC-only and RPTEC/HUVEC systems. Four groups are presented, namely, (i), RPTECs on D14 in the culture dishes; (ii), RPTECs of the bilayer under static culture condition; (iii), RPTECs of the bilayer under perfused culture conditions; (iv) Single layer RPTECs under perfused media. Proximity with HUVECs under media perfusion has augmented the expression levels of *AQP1*, *EpCAM*, *MDR1*, *OCT2*, and *SGLT2* (compare groups iii and iv). Independently, perfusion has increased the expression levels of the same genes (with the possible exception of *SGLT2*) in the co-culture (compare groups ii and iii). *n* = 5 PCR replicates were analyzed for each gene/sample obtained from one set of experiment. **c** Transfer rates of the glucose probe, 2NBDG measured in static and perfused culture conditions. Both reabsorption (a → b) and reverse transfer rates (b → a) were quantified. The inhibitory effect of phlorizin (P.) on glucose transport was also examined; *n* = 3 independent chips. **d** Apparent permeabilities to Rh123 in both excretion (b → a) and reverse (a → b) directions. Verapamil (V.), a contender for Rh123 extrusion, was applied to attenuate Rh123 efflux and confirm the function of Pgp; *N* = 3 independent chips. **e** Fluorescent confocal z-stacked images of the RPTEC tissue layer in bilayer and single layer configurations. A considerably higher amount of BSA was precipitated in the basolateral milieu of RPTECs in the bilayer system indicating a higher intake of the substrate. Some BSA is precipitated in the single layer RPTEC on D17 (white arrowhead). Scale bar is 50 μm. **f** Transport rates of AF488-conjugated bovine serum albumin (BSA-AF488) in both directions. In this study, we examined the effect of reducing the incubation temperature (to 4 °C) on selective transport of BSA-AF488; minimum *N* = 6 independent chips. In (**b**–**d**, **f**) two-sample t-test was run between pair of data sets as indicated. Error bars represent standard deviation; N.D., not detected; N/A, not available; N.S., not significant; *, **, ***, ****, for *p* ≤ 0.05, 0.01, 0.001, and 0.0001, respectively. a → b, apical to basal; b → a, basal to apical.

The protocol for the formation of the RPTEC/HUVEC bilayer is once again depicted in Fig. 7a. To determine whether the presence of HUVECs would affect markers of proximal tubule structure and function, we conducted qPCR analysis. Apart from morphological improvements observed in the microvillar apical membrane, we noticed mRNA levels of certain RPTEC genes are increased under flow induced shear stress and in the presence of HUVECs. On the one hand *AQP1*, *EpCAM*, *MDR1*, *OCT2*, and possibly *SGLT2* are upregulated in the mature bilayer system subjected to perfusion flow, on the other hand vicinity with HUVECs under media perfusion has caused the expression levels of *AQP1*, *EpCAM*, *MDR1*, and *OCT2* to increase (Fig. 7b).

Thus, we expected that the epithelium brought to the proximity of HUVECs may exhibit not only a denser structure but also higher xenobiotic elimination and glucose uptake rates. These suppositions were confirmed by in-depth assessments of glucose reabsorption and Rh123 excretion rates, indicating that the level of proteins corresponding to *SGLT2* and *MDR1* (SGLT2 and Pgp) were enhanced as well (Fig. 7c, d). The a → b glucose reabsorption rates were significantly improved in presence of HUVECs and under perfusion flow, independently, whereas the b → a rates were almost unaltered. Also, phlorizin as a classic competitive inhibitor of both *SGLT1* and *SGLT2*^41–43^, effectively blocked reabsorption of glucose.

Although LRP2 was not stably detected, presence and proper function of megalin was confirmed in RPTECs by analyzing albumin reabsorption rates. We showed that the albumin uptake capacity of RPTECs increases considerably in the presence of HUVECs. This is evidenced by accumulation of BSA in the basolateral side of RPTECs in the bilayer system (Fig. 7e). In addition, the bilayer displayed a higher rate of albumin a → b transcytosis (Fig. 7f). Lowering temperature during the measurements seems to have considerably reduced reabsorption rates as investigated previously^44,45^.

These results are interesting, particularly because the reabsorption and excretion rates are increased even though, intuitively, the endothelial layer may hinder the transfer of any biomolecule across the channels. Paracrine signaling between the two cell types has improved megalin, SGLT2, and Pgp function to the extent that glucose, albumin, and Rh123 are all transferred at considerably higher rates across the bilayer tissue. In addition, apical microvilli have become denser in the RPTECs cocultured with HUVECs even in the absence of mechanical stimuli (Fig. 5k). Notice that in the RPTEC/HUVEC bilayer system there was no tangible improvement in the overall efflux ratio of Rh123, whereas the glucose and albumin reabsorption rates were both increased. Lastly, we evaluated the capacity of the basolateral xenobiotic transporter, OCT2, in the single layer and bilayer systems. OCT2 is responsible for the uptake of chemotherapeutic drugs such as oxaliplatin and cisplatin that are known to cause drug-induced kidney injury^46^. Using cimetidine as an OCT2 inhibitor we showed selective uptake of cationic fluorescent EtBr and vectorial uptake of 4-Di-1-ASP (ASP+), both known as OCT2 substrates (Supplementary Fig. 7). However, in contrast to albumin, glucose, and Rh123 filtration assays, where HUVECs proved to improve the transport rates in the bilayer, the RPTEC single layer was more efficient in absorbing ASP+ as the substrate was blocked by HUVECs.

## Discussion

We report engineering of a 2D proximal tubule tissue by combining cells extricated from hiPSC-derived kidney organoids (LTL+) with differentiated human RTPEC/TERT1 cells. The contrived tissue layer displayed enhanced filtration/reabsorption capacity compared with its non-iPSC-based counterpart. In addition, mRNA expression levels of certain proximal tubule-specific genes were amplified in the coculture signifying higher degrees of maturity and function. Our engineered epithelium, comprised of perfectly merged kidney organoid-derived and immortalized cells, could stably maintain its conformality at least up to day 14.

As outlined in the protocol, during the intermediate mesoderm differentiation the ratio of metanephric mesenchyme to ureteric epithelium progenitors can be adjusted by choosing the day of CHIR-to-FGF9 switch^19^. A later switch date will produce more of the former, thus giving rise to the cap mesenchyme and leading to more nephrons. Since we are merely interested in harvesting proximal tubule-like cells we chose the later date of switch (day 5). The substantial increase in the expression level of K-cadherin observed after 2D culture, reported earlier, can be attributed to the higher proliferation rate of CDH6-high, LTL-low cells, known to serve as proximal tubule progenitors, as compared to the more mature CDH6-low, LTL-high differentiated population^47^.

In parallel, we revisited the literature on 2D proximal tubule models by examining and highlighting the effects of endothelial cells and flow-induced shear stress on the characteristics and performance of mature contact-inhibited proximal tubule epithelial layers developed on PET membranes. It was established that HUVECs significantly improve the intensity and appearance frequency of tight junctions, mRNA levels of *AQP1*, *EpCAM*, *OCT2*, *MDR1*, and *SGLT2*, and the vectorial transport rates of respective substrates, glucose, and Rh123. Supplementary Tables 2 and 3 summarize the improvements in glucose uptake rates and Rh123 efflux ratios in the bilayer and coculture systems over the RPTEC-only case. In contrast to LTL+ cells, *LRP2* was undetected (or merely found in miniscule amounts) in RPTECs, confirming the previous studies^15^. However, megalin whose amount could be modulated by perfusion flow was abundantly expressed. We also demonstrated and measured the rates of albumin transport. Although the rates obtained here are far less than the cases in vivo, it is apparent that most of the mass transfer is hindered by the membrane.

To the best of our knowledge, no previous study has incorporated proximal tubule cells derived from hiPSC-based kidney organoids, independently. Nor has anyone attempted to construct a 2D functional tissue by coculturing hiPSC kidney organoid-derived epithelial cells with immortalized cell lines. Others have generated and characterized proximal tubule-like cells by directed differentiation of iPSCs, first generating cells of the intermediate mesoderm (IM) and then driving the IM cells into renal cells^15^. Their differentiation protocol generated cluster of cells resembling early stages of renal development and were stable for up to 7 days. Typical markers such as *CDH6* and *LRP2* were detected expressing mRNA levels with a similar pattern explored herein, whereas there were no traces of *ABCB1*. On the contrary, we detected the gene at a low level in our LTL+ cells. While no major difference was observed in the expression level of *ABCB1* between RPTEC and coculture tissues, the protein level and activity were significantly improved in the coculture. Hence, we agree with and confirm the notion that the Pgp protein/activity does not necessarily follow its gene expression levels. In this previous work, although Pgp function was demonstrated qualitatively by showing accumulation of calcein-AM in the presence of a competitive Pgp inhibitor, Cyclosporine A (CsA), quantitative transport data such as the efflux rates were left unprobed.

We believe that our coculture system offers a few advantages over existing models of the proximal tubule epithelium. For example, it enables patient-specific disease modeling, drug screening, and pathogenesis study, by incorporating pertinent patient-derived iPSCs to form the organoids. These organoids can then be subjected to the same procedure outlined here to obtain the diseased cells that can be cocultured with cell lines to eventually form a homogenous tissue for analysis and treatment purposes. Moreover, the concept of mixing cells obtained from iPSC-derived organoids with immortalized cells to facilitate or hasten secretion of ECM and form a 2D functional tissue may offer ubiquitous applications in tissue engineering.

During cell sorting experiments we realized minor modifications in the protocol can optimize the efficiency of harvesting the positive fraction in particular. Variability in the number of live cells dissociated from multiple organoids markedly affected the MACS success rate but this could be addressed by adjusting the concentration of antibodies. The fact that the semi-iPSC-based tissue forms an epithelial barrier more rapidly that the contender was demonstrated through our robust four-probe TEER measurement technique. Our choice of protocol to generate kidney organoids was not haphazard. At the time we instigated the concept of extracting iPSC-derived cells from kidney organoids, the protocol put forward by Takasato et al.^19^ generated the most complex hiPSC-derived kidney organoids. Later advances in the field demonstrated kidney organoids with possibly more intricate nephron structures^14^.

Although the PET membrane considerably impedes transport through the bilayer, we could quantify transport rates in various conditions and demonstrate vectorial transport of albumin, glucose, and Rh123 with the ability to modulate each by relevant classic inhibitors. Sufficient epithelium-endothelium crosstalk through the membrane led to improved tissue integrity (tight junctions), denser apical microvilli, and gene expression levels particularly those of transporters, *MDR1*, *OCT2*, and *SGLT2*.

The key difference between our method and the previous protocols^6,7^ is our strategy to form/investigate an already mature (contact-inhibited) epithelial proximal tubule layer prior to introducing endothelial cells to the equation. In addition, we refrained from applying any additional (artificial) ECM coating to the membrane allowing cells to secrete their own ECM. The tissue construct was deliberately meant to be shaped flat to facilitate characterization of the coculture and quantitative analysis of relevant reabsorption/filtration rates in a microfluidic chip.

## Methods

### Microfabrication

The chips are comprised of two identical PDMS slabs and a porous PET membrane sandwiched in between (Supplementary Fig. 3a). PET membranes having a pore size and porosity of 3.0 μm and 5%, respectively, were cut off Falcon permeable supports (Corning 353091). The slabs were replica molded on ¼ of 4” Si(100) wafers carrying embossed microfluidic channel patterns formed using standard SU-8 photolithography. Briefly, a 10:1 mixture of PDMS (Sylgard 184, Toray Dow Corning) and curing agent was poured on the molds positioned in petri dishes. After curing and detaching the slabs from the molds we examined two methods to mount and bond the membrane. In the first approach, a very thin layer of uncured PDMS was spun on the bottom slab to serve as an adhesion layer on which the membrane was positioned. The top slab was then placed on the membrane and carefully aligned under microscope so that the microfluidic channels, separated by the membrane, would completely overlap. The chip was finally cured in an oven at 65 °C for 1 h. In this fashion, the slabs could be easily and precisely aligned before the curing step; however, the membrane was deformed at high curing temperature. In addition, some of the uncured PDMS diffused into the channels reducing the available area of the membrane for cell culture. Devices fabricated in this fashion were used for preliminary validation experiments, including cell culture and immunostaining. In the second method, no adhesion layer was applied. Briefly, the membrane and the bottom slab with microchannel side up were treated under O_2_ plasma (Covance-1MP, Femto Science Inc.) for 1 min to render the surfaces hydrophilic. The membrane was then placed on the bottom slab. Next, the top slab along with the membrane-bearing bottom slab were exposed to O_2_ plasma for 1 min. The slabs were immediately brought together and bond under microscope. In this manner, there was a one-shot chance to align the microchannels under microscope as oxidized PDMS slabs would strongly bond to each other after being in contact. Nevertheless, because all the process was carried at room temperature the membrane remained perfectly flat. After bonding, 2 mm wide holes were punched through the slabs at both ends of the microchannels. In general, we did not notice any major deviation in the measured results that could be attributed to the slight membrane curvature or reduction of its apparent area. However, devices fabricated at room temperature were used in the quantification of transport rates.

### Cell culture and seeding onto the PToCs

#### hiPSC maintenance and culture

hiPSCs were passaged three times before seeding for differentiation. After thawing, all three passages were carried in 6-well plates. Plates were first coated with iMatrix-511 (0.5 mg mL^–1^, Nippi, 892011/892012) at 3 μg mL^–1^ prepared in 1 mL DPBS per well (1/6). Plates were incubated at 37 °C for 1 h, then filled with 0.75 mL (per well) of StemFit (Ajinomoto, AK02N) containing 10 μM of ROCK inhibitor (Y27632, 10 mM, Wako, 253-00511) and incubated again. Cryovials containing 10^6^ cell mL^–1^ of unlabeled hiPSCs (female CRL1502-C32 fibroblasts derived from ATCC CRL-1502 fetal fibroblasts^48^) or labeled hiPSCs (H2B-GFP-integrated 1502.3) frozen in 200 µL of Stem Cell Banker were rapidly thawed in a 37 °C water bath. The cells were carefully transferred to 5 mL Eppendorf tubes containing 5 mL of StemFit using wide bore 1 mL pipette tips. The tube was centrifuged at 200 G for 5 min. After aspirating the supernatant, the cells were resuspended in 100 μL of StemFit and counted. A 1.5 mL cell suspension was prepared in StemFit for each well in a fashion to contain 10^4^ cells and added to iMatrix-coated well plates. After seeding, the plates were kept at room temperature for 5 min to maintain a homogenous coverage throughout the well and then incubated at 37 °C. In between passages and prior to dissociation, cells were washed with DPBS (1 mL per well) two times, soaked in TrypLE (Fisher, 12563-029) 0.3 mL, incubated for 5 min, agitated by tapping once, and incubated again for 3 min. Cells were collected from each well into 5 mL tubes by adding 1 mL of StemFit and reseeded on iMatrix-coated well plates at 10^4^ cell per well as described above. After three times of passaging, cells were dissociated and cultured on day –1. Differentiation was commenced on day 0 as outlined in ref. ^19^. In brief, the posterior primitive streak was induced by activating bone morphogenetic protein (BMP) and canonical WNT signaling using glycogen synthase kinase (GSK-3) inhibitor, CHIR99021, in APEL. CHIR was switched to FGF9 on day 5 to induce intermediate mesoderm. On day 7, 3D self-organization was initiated by culturing aggregates on transwell filters. Growth factors were withdrawn on day 12.

#### RPTEC/TERT1 culture

Cryopreserved immortalized RPTEC/TERT1 cells (ATCC® CRL-4031™) stocked at 10^6^ cell/vial with passage number less than 9 were thawed and subcultured in T25 cell culture flasks until they reach ∼90% confluency. During the subculture period that lasted 5 days, DMEM/F12 (Gibco 11320033) supplemented with hTERT immortalized RPTEC growth kit (ATCC® ACS4007™) was used as culture medium per manufacturer’s instructions. The cells were trypsinized and resuspended at 4 × 10^6^ cell/mL in REGM™ (Lonza CC-3190). The chip microchannels and reservoirs were sterilized with 70% ethanol, dried under clean bench, and exposed to UV light for 1 h. Prior to seeding, the membrane was coated with a cell adhesion agent, FNC Coating Mix® (Athena 0407), and incubated at 37 °C for 1 min to enhance the rate of cell attachment. After filling the bottom channel of each device with 200 μL of REGM, 30 μL of the cell suspension was gently injected into the top channel. The devices were incubated at 37 °C, in 5% CO_2_ for 1 h until the RPTECs completely settle and attach to the membrane, after which 170 μL of REGM was added to the top channel. The devices were maintained in the incubator and monitored while the media in both channels were refreshed on a daily basis.

#### HUVEC culture

On chips designated to carry the bilayer, RFP-HUVECs were cultured on the opposite side of the membrane on day 10. Briefly RFP-labeled HUVECs (Angio-Proteomie Co. cAP-0001RFP) stocked at 5 × 10^5^ cell/vial with passage number less than 4 were subcultured in EGM2™ (Lonza CC-3162) in a similar fashion to RPTECs. Upon confluency (∼90%) cells were resuspended at 4×10^6^ cell/mL in EGM2. After aspirating REGM, 30 μL of the HUVEC suspension was gently injected into the bottom channel of each device. The devices were flipped immediately and incubated for 2 h until the endothelial cells attach properly. The devices were then flipped back and 170 μL of EGM2 was added to the bottom channel. Thereafter, REGM and EGM2 were replaced every day.

### Perfusion culture

We designed a custom made perfusion culture system setup to operate at 37 °C, 5% CO_2_ and capable of handling six devices simultaneously. Starting from day 11/day 1 of RPTEC/HUVEC culture, REGM and EGM2 from sterilized glass jars containing 3 mL of each were perfused both at either 10 μL min^–1^ or 1 μL min^–1^ through the corresponding microchannels to mimic culture conditions under static and flowing media, respectively. At a flow rate of *Q* = 10 μL min^–1^ the shear stress exerted upon the apical membrane of epithelial cells, *τ*, was estimated to be 0.06 dyn cm^−2^ using

1 $$\tau =6\,\eta \,Q\,{W}^{-1}\,{h}^{-2},$$

where *η* = 0.7 is the approximate medium viscosity at 37 °C, and *h* = 0.35 mm and *W* = 1.0 mm are the channel height and width, respectively. The entire media in the jars was replaced every other day.

### Immunofluorescence of cell-laden membranes

Cells on the either side of the membrane were fixated by applying 4% paraformaldehyde in DPBS (1X) (Gibco®) into both channels for 10 min, permeabilized with 0.1% Triton X-100 for 10 min unless otherwise noted, and then soaked in blocking buffer of 10% donkey serum for 1 h, all at room temperature. Mixtures of primary antibodies prepared in the blocking buffer were applied into both microchannels and the samples were left at 4 °C overnight. In the following day, membranes were thoroughly rinsed with DPBS three times 30 min each, then soaked in the mixture of secondary antibodies, prepared in DPBS, for 1 h at room temperature. The membranes were then treated with DAPI for 10 min, followed by an antifade mountant (Fisher, S36937) and mounted on microscope slides. Confocal fluorescent microscopy was performed with an Olympus FV3000 microscope. Images were analyzed by FV31S-SW (v2.5.1.228, Olympus) viewer software and Image J (v1.53f51, NIH, USA). Supplementary Table 4 lists the concentrations of antibodies used.

### Transmission electron microscopy

Cell-laden membranes in the chips were rinsed with DPBS first, fixated overnight at 4 °C with a buffer consisting 2% glutaraldehyde (EM grade, Electron Microscopy Science) and 0.1 M NaPO_4_ in water, and subsequently post-fixed in 2% OsO_4_ (Crystal, Heraeus Chemicals, South Africa) for 3 h in an ice bath. The chips were outsourced for microscopy. Upon arrival at the facility, the specimens were dehydrated in graded ethanol (50, 70, 90, 100, 100, and 100%, each for 15 min) and embedded in epoxy resins. Ultrathin 80 nm sections were cut by ultramicrotome technique. Ultrathin sections stained with uranyl acetate for 10 min and lead staining solution for 5 min were analyzed on a HITACHI H-7600 at 100KV.

### Quantitative RT-PCR

Total mRNA was extracted and purified from 3 individual samples per condition using RNA isolation kit (NucleoSpin® RNA). We used a maximum amount of 3 μg/60 μL of mRNA for reverse transcription with PrimeScript™ RT Master Mix kit as per the manufacturer’s instructions. Template cDNA samples were diluted (1:10) in TaqMan® Universal PCR Master Mix and TaqMan® Gene Expression Assay forward and reverse primers per gene of interest and distributed into 5 wells of a 384 multi-well reaction plate. Quantitative RT-PCR was performed using the QuantStudio® 5 Real-Time PCR detection system (ThermoFisher Scientific). β-actin was used as the endogenous gene. Primer data are listed in Supplementary Table 5.

### FACS

30 kidney organoids (KOs) were prepared following the protocol of Takasato et al. (2016). The organoids were harvested on D22 of induction and dissociated using the enzymes provided in the Tumor Dissociation Kit (Miltenyi Biotec, #130-095-929) as per the manufacturer’s instructions. The triple enzymes, H, R, and A, as prepared, were dissolved in a separation buffer at concentrations of 4, 2, and 0.5% (v/v), respectively. The separation buffer was comprised of DPBS, EDTA (Fisher, 15575020) at 2 mM and FBS at 0.5% (v/v). Dissociated cells were suspended in the same buffer throughout the separation and sorting processes up to the seeding stage. Sorting was performed using a BD FACSAria™ III cell sorter and the sorted cells were resuspended in REGM containing 10 μM of ROCK inhibitor (Fuji Film, CultureSure® Y-27632) for seeding or mRNA isolation. See Supplementary Fig. 1, 2 for more details.

### MACS

For each experiment 16 KOs were prepared. The organoids were harvested between D22-26 of induction, dissociated in a similar fashion as in FACS, suspended in the separation buffer and preserved on ice throughout the entire process prior to sorting. The samples were briefly centrifuged, resuspended, and divided into two equal portions (8 KO per portion). Each portion was passed through a 10 µm pluriStrainer® (pluriSelect, #43-50010-03) sieve to remove any remaining cell clusters. The sieved portions were remixed, centrifuged, resuspended in separation buffer, and counted. Biotinylated *lotus tetragonolobus lectin* (LTL), a robust proximal tubule brush border marker, was applied to the suspension at a 1:100 ratio as the primary antibody. After incubating for 30 min at 4 °C the sample was centrifuged and resuspended two times to completely remove any unattached antibody. Monoclonal Anti-Biotin MicroBeads (Miltenyi Biotec, #130-105-637) were applied at a 1:5 ratio for indirect magnetic labeling. The suspension was then incubated at 4 °C for 30 min, centrifuged and resuspended in separation buffer (500 μL). The suspension was gently applied to an MS Column (Miltenyi Biotec, #130-042-201) plugged into one of the eight slots of an OctoMACS separator (Miltenyi Biotec, #130-042-108). Before applying the suspension, a 30 µm Pre-Separation filter (Miltenyi Biotec, #130-041-407) was placed on the column to remove any clumps that would otherwise clog the pores and the entire pillar was wetted with 500 µL of DPBS. After the suspension was applied, the column was rinsed with 500 µL of separation buffer two times to completely deplete the negative fraction (LTL–) (total volume 1.5 mL). To collect the positive fraction (LTL+) the column was removed from the separator, placed on a collection tube, and immediately flushed with 1 mL of separation buffer. MACS success rate refers to the sum of final MACS product (LTL+ and LTL– cells) divided by the total number of filtered live cells (< 10 μm) that were subjected to LTL marker, microbeads, and subsequently applied to the separation columns. Evidently, 50% of the cells were lost during tagging with antibodies. During the course of optimization experiments, we had established that by decreasing the concertation of LTL antibody in the cell suspension from 1:50 to 1:100 the specificity would increase by 7%. The charts in Fig. 1c show the relative populations of final MACS products as a function of LTL antibody concentration. Supplementary Table 1 shows the statistics of the MACS process. Data are collected from seven consecutive experiments. Finally, both fractions were resuspended in REGM containing 10 μM of ROCK inhibitor for seeding or mRNA isolation.

### Albumin reabsorption assay

Small-volume aliquots of AF488-conjugate BSA (ThermoFisher, A13100) were prepared at 5 mg mL^–1^ and stored at −30 °C until usage. Using our homemade perfusion system, regardless of the tissue configuration (single layer or bilayer) we circulated 3 mL of REGM containing 50 μg mL^–1^ of AF488-conjugate BSA (ThermoFisher, A13100) and pristine EGM2 through the top and bottom channels, respectively. Media were perfused and circulated for 24 h. 10 μL samples were collected from the EGM2-containing reservoir sourcing the bottom channel at 6-h intervals. The diffusate concentrations in EGM2 were measured immediately using a spectrophotometer (NanoDrop™ 3300). It was assumed that removing volumes of 10 μL 4 times from a 3 mL source does not disturb the diffusion equilibrium. We established that the circulation of media in both concentrate and diluate channels is necessary to maintain a sufficient albumin concentration gradient. Because of its high molecular weight (66 kDa), only a minuscule amount of BSA could diffuse in the static case (Supplementary Table 6). The reverse basal to apical transfer was quantified in a similar fashion, but instead AF488-conjugate BSA was dissolved in EGM2 supplying the bottom channel and measured in pristine REGM at the top. To assess the inhibitory effect of lowering the temperature on albumin reabsorption, the setup was placed in a 4 °C dark room instead of the incubator.

### Glucose reabsorption assay

Stock solutions of 2-NBD-Glucose, fluorescent glucose uptake probe (2-NBDG, Abcam, ab1462002) were prepared at 30 mM and stored at –30 °C for later use. After rinsing off the media, 200 μL of DPBS+ (Gibco, 14040133) was applied to the top and bottom channels and then the devices were soaked at 37 °C for 30 min. Glucose reabsorption measurement began by replacing 100 μL of DPBS+ at the top channel with the same volume of 1 mM 2-NBDG solution to achieve a final concentration of 0.5 mM. At 1-h intervals the diffusate at the bottom channel was mixed gently, yet thoroughly, by manually circulating (pipetting) a volume of 100 μL two times. The same amount was sampled and stored in dark Eppendorf tubes. Depleted buffer was replenished immediately by adding 100 μL of fresh DPBS+. Sample concentrations were measured in between sampling intervals by NanoDrop™ 3300. We computed the time course concentration of glucose from the concentration of samples taken at each interval by reflecting the eliminated amount, using the following equation:

2 $${C}_{{{{{{\rm{t}}}}}}}={C}_{{{{{{\rm{t}}}}}}-1}+({R}_{{{{{{\rm{t}}}}}}}\times 200\,{{{{{\rm{\mu }}}}}}{{{{{\rm{L}}}}}}-{R}_{{{{{{\rm{t}}}}}}-1}\times 100\,{{{{{\rm{\mu }}}}}}{{{{{\rm{L}}}}}})/1000\,{{{{{\rm{\mu }}}}}}{{{{{\rm{L}}}}}},$$

where *C*_t_ and *C*_t–1_ represent actual concentrations of 2-NDBG in the channel at current and previous sampling time points, respectively, and *R*_t_ and *R*_t – 1_ are the corresponding sample concentrations all in nmol min^–1^. The volumes 200 μL and 100 μL refer to the total channel and sampling volumes, respectively. Rates were obtained by conducting linear regression on the time course concentration data over 0 ≤ *t* ≤ 3 h. SGLT inhibition was evaluated by administering phloridzin. Phloridzin (P3449, Sigma) aliquots were prepared in 70% ethanol at 100 mM and stored at –30 °C for later use. The drug was applied at a concentration of 1000 μM (100× dilution). Vectorial exocytosis of glucose was examined by quantifying the mass transfer rates at the reverse direction, i.e. basal to apical. In this case 2-NBDG was applied to the bottom channel and measured at the top.

### Rhodamine 123 excretion studies

We examined the performance of Pgp efflux pump by measuring the transfer rate of Rh123 (Fisher, R302) applied at 2.5 μM. To inhibit Rh123 efflux, we added verapamil as a Rh123 contender at 300 μM. After removing and rinsing off culture media, the top and the bottom channels were filled with 200 μL of HBSS pH 6 and HBSS pH 7.4, respectively. Devices were preincubated at 37 °C for 30 min. Sampling was carried out in a similar fashion to the glucose reabsorption measurement procedure. However, since the efflux (basal to apical) rate was desired, the substrate was mainly applied to the bottom channel and measured at the top. Briefly, to maintain a source concentration of 2.5 μM, 100 μL of the buffer from the bottom channel was replaced with 5 μM solution of Rh123 prepared in HBSS pH 7.4, w/ or w/o verapamil. 100 μL samples were collected from the top channel at intervals and transferred into individual wells of a black bottom 96-well plate (Corning, CLS3925). Each well was pre-filled with HBSS to achieve a working volume of 200 μL as suggested by the microplate reader’s manufacturer. The top channel was topped up with 100 μL of HBSS pH 6 immediately after sampling. Prior to sampling, a single row of the 96-well plate was filled with Rh123 solutions of known concentrations to obtain calibration data. The fluorescence emission of samples was measured by a microplate reader (Molecular Devices, SpectraMax® iD5). To quantify the transfer rates at the reverse direction, Rh123 (w or w/o verapamil) was dissolved in HPSS pH 6 and administered from the top channel.

Calibration data of all the fluorescent substrates are presented in Supplementary Table 7.

### EtBr and ASP+ uptake studies

For the ethidium bromide (EtBr) uptake experiments the control samples consisted of only HUVEC monolayers cultured on the top layer. In a similar manner to the Rh123 assay, all samples were preincubated in acidic and neutral HBSS buffers. As cationic fluorescent probes, we introduced 2.5 μM of EtBr to the bottom channel (basal side for RPTECs) or 2.5 μM of 4-Di-1-ASP (ASP+) into apical and basal side, separately. After introduction of the probes, the devices were incubated for 4 h. RPTEC and HUVEC monolayers were then imaged using an inverted fluorescent microscope at 60 min and 30 min intervals for EtBr and ASP+ experiments, respectively. We concurrently administered 1 mM of cimetidine as an OCT2 inhibitor to verify the transporter-dependent uptake of EtBr and ASP+. Relative fluorescence intensities of samples were computed from individual snapshots using Image J software and normalized to that of the RPTEC w/o inhibitor.

### TEER measurements

Measurements were carried out in microfluidic chips using a custom-made four-probe setup^28^. Briefly, RPTECs or RPTEC/LTL+ cell admixture was cultured on the top layer of the membrane as described previously. The impedance of the cell-laden membranes was measured using an LCR meter (ZM2731, NF Co.) with sinusoidal stimulus over a frequency range of 100 Hz to 10 kHz under a peak-to-peak voltage of 20 mV. The baseline impedance was recorded prior to seeding, with both channels filled with REGM. TEER values were obtained by subtracting the baseline resistance from the resistances as measured. All measurements were performed in an incubator at 37 °C, 5% CO_2_. The four-probe technique allows us to eliminate series resistances of the culture medium and the electrical double layer on the surface of electrodes. We applied a fast Fourier transform (FFT) low pass filter to smooth the TEER curves with a cut-off frequency of

3 $${f}_{3{{{{{\rm{dB}}}}}}}=1/2\,n\,\varDelta t=0.167\,{{{{{{\rm{day}}}}}}}^{-1},$$

with points of window value of *n* = 6, and time interval of Δ*t* = 0.5 day.

### Statistics and reproducibility

In general, a total of *N* = 3 independent devices (chips) per condition were used to collect measurement data (transport rates, etc.), unless noted otherwise. To examine the significance between two group of data, two-sample t-test was performed with significance level set to 0.05. We excluded the statistical significance analysis on qPCR data of Fig. 4a as there were only *N* = 2 independent experiments for RPTEC and LTL+ cases. The qPCR data presented in supplementary information (i.e. Supplementary Figs. 1d, 2b) are collect from one experiment per condition with *n* = 5 technical replicates. Hence, no statistical analysis was conducted among the groups. Other details are in are explained in figure captions.

### Reporting summary

Further information on research design is available in the Nature Portfolio Reporting Summary linked to this article.

## Supplementary information

Supplementary Information

Description of Additional Supplementary Files

Supplementary Data 1

Supplementary Movie 1

Supplementary Movie 2

Supplementary Movie 3

Supplementary Movie 4

Supplementary Movie 5

Supplementary Movie 6

Supplementary Movie 7

Supplementary Movie 8

Supplementary Movie 9

Supplementary Movie 10

Supplementary Movie 11

Reporting Summary

## Data Availability

Numerical source data for graphs and charts are stored at 10.6084/m9.figshare.22339615.v2.
