# Supplementary material for: Cells sorted off hiPSC-derived kidney organoids coupled with immortalized cells reliably model the proximal tubule
Source: Commun Biol. 2023 May 4;6:483. doi: 10.1038/s42003-023-04862-7 (PMC10160057; doi:10.1038/s42003-023-04862-7)
Supplement: Supplementary file 1 — Supplementary Information [file 42003_2023_4862_MOESM1_ESM.pdf]

Cells sorted off hiPSC-derived kidney organoids coupled with immortalized cells reliably model the proximal tubule

Supplementary Information

Ramin Banan Sadeghian[1], *Ryohei Ueno*[1], *Yuji Takata*[1], *Akihiko Kawakami*[1], *Cheng Ma*[1],

Toshikazu Araoka[2], *Minoru Takasato*[3,4,5], *and Ryuji Yokokawa*[1]*

[1] Department of Micro Engineering, Kyoto University, Kyoto, 615-8540, Japan
[2] Center for iPS Cell Research and Application (CiRA), Kyoto University, Kyoto, 606-8507, Japan
[3] RIKEN Center for Biosystems Dynamics Research (BDR), Kobe, 650-0047, Japan
[4] Graduate School of Medicine, Osaka University, Osaka, 565-0871, Japan
[5] Graduate School of Biostudies, Kyoto University, Kyoto, 606-8501, Japan

*Email: yokokawa.ryuji.8c@kyoto-u.ac.jp

Keywords: proximal tubule, microphysiological systems, hiPSC-derived kidney organoids, filtration, reabsorption

Contents

1. **Supplementary Table 1**| MACS statistics for N = 7 experiments.
2. **Supplementary Figure 1**| Attempts to characterize PT-like cells extracted from hiPSC-derived kidney organoids (KOs) through FACS, using a single marker (LTL).
3. **Supplementary Figure 2**| Attempts to characterize PT-like cells extracted from hiPSC-derived KOs through FACS using two markers (LTL and megalin).
4. **Supplementary Figure 3**| Construction of the Proximal Tubule on a Chip (PToC).
5. **Supplementary Figure 4**| Some details pertinent to the perfusion culture system and appearance of confluent tissue layers on the PToC.
6. **Supplementary Figure 5**| Exemplifying how protein-to-nucleus distance is computed from z-intensity profiles.
7. **Supplementary Figure 6**| High magnification TEM snapshots of RPTECs.
8. **Supplementary Figure 7**| Demonstration of OCT2 function in single layer and bilayer RPTEC tissue constructs.
9. **Supplementary Table 2**| 2NBDG uptake (a $\rightarrow$ b) rates in µg h^{-1} in various conditions and in the absence of SGLT2 inhibitor.
10. **Supplementary Table 3**| Rh123 efflux ratios, P_{app} (b $\rightarrow$ a) / P_{app} (a $\rightarrow$ b), in various conditions and in the absence of Pgp inhibitor.
11. **Supplementary Table 4**| List of antibodies used and their concentrations.
12. **Supplementary Table 5**| List of TaqMan primers used for qPCR analysis.
13. **Supplementary Table 6**| BSA-AF488 diffusion rates compared between membranes with no cell and those with HUVEC only layers, in static and perfused cases.
14. **Supplementary Table 7**| Calibration details for the fluorescent substrates used in this study.

Data

1. **Supplementary Data 1**| Flow cytometry cell population data pertinent to Supplementary Fig. 1 & 2

Videos

1. **Supplementary Movies 1, 2**| Proximal tubule-like cell populations in hiPSC-derived kidney organoids.
2. **Supplementary Movies 3-6**| Comparison of proximal tubule cells from different sources.
3. **Supplementary Movies 7-9**| High magnification confocal laser scans through the coculture tissue
4. **Supplementary Movies 10, 11**| Construction of the bilayer tissue.

Supplementary Table 1 | MACS statistics for N = 7 experiment. μ , mean; δ, standard deviation.

	Cells per organoid		Cells < 10 μm per organoid		MACS success rate (%)	Specificity LTL+ / all labeled cells (%)
	No. (live)	Viability (%)	No. (live)	Viability (%)		
μ	6.46×10^5	91	3.88×10^5	90	51.7	37.5
δ	1.53×10^5	5	9.54×10^4	4	15.0	16.0

- MACS success rate is defined as the sum of LTL+ and LTL– cell numbers divided by the total number of dissociated cells that were passed through a 10 µm sieve.

- Specificity is defined as the number of LTL+ cells divided by the total number of cells marked with micro-beads (see the Methods section).

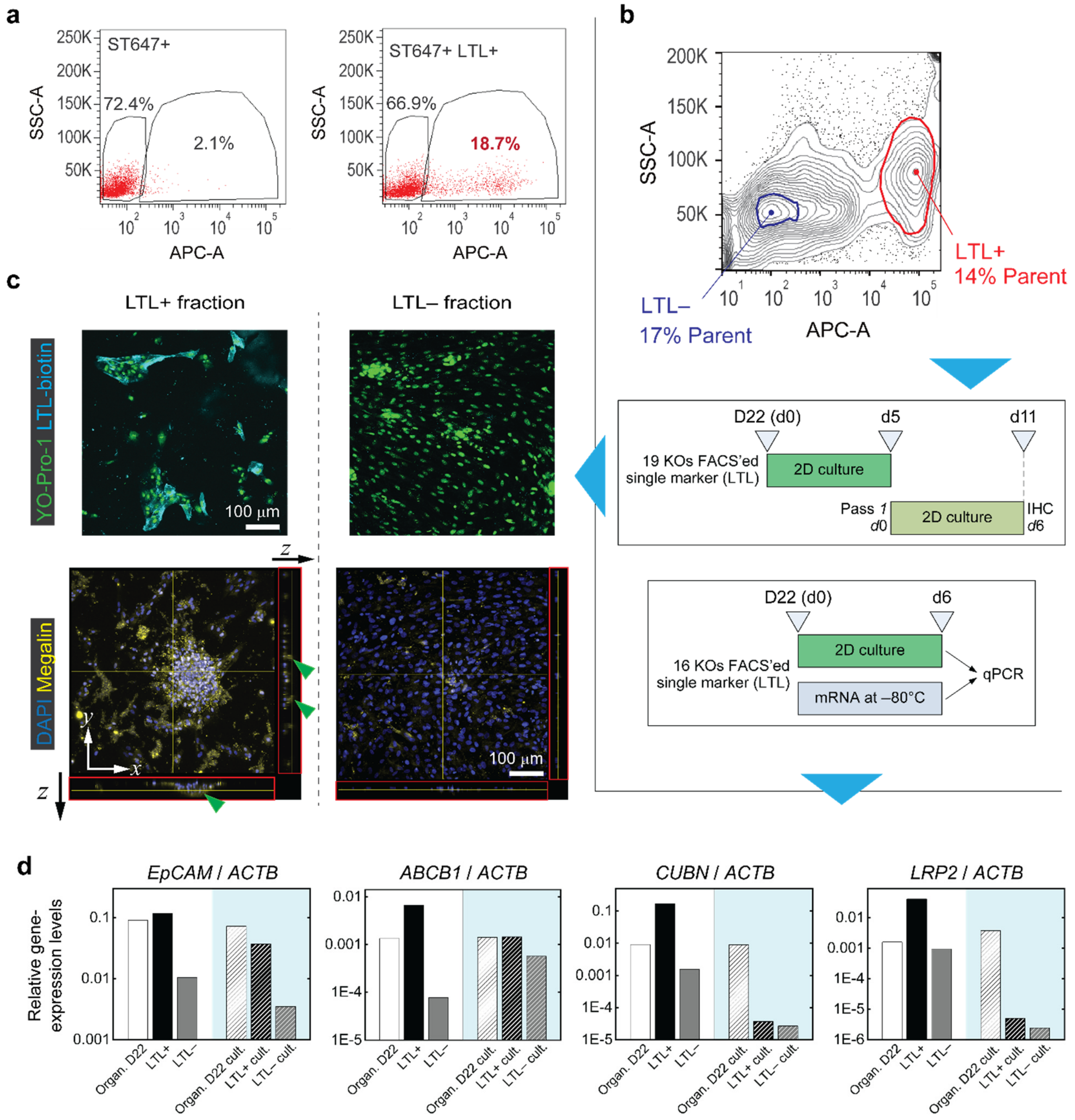

Supplementary Figure 1| Attempts to characterize PT-like cells extracted from hiPSC-derived kidney organoids (KOs) through FACS, using a single marker (LTL). (**a**) Flow cytometry plots showing the gating strategies applied to distinguish the positive fraction of labeled cells (LTL+). Alexa Fluor 647 Streptavidin (1/500) was used as the secondary antibody. The left plot corresponds to the control experiment (with secondary antibody only), whereas the right plot shows the distribution of actual LTL+ cells (18.7%). Evidently, FACS provides a higher specificity than that of MACS (37.5% as shown in Supplementary Table 1). (**b**) Flow cytometry contour plot representing the densities of LTL-positive/negative (LTL+/–) cell populations for cells sorted off KOs that were harvested on D22. The gating strategies for the cells to be sorted are shown. Cells were subjected to IHC or qPCR as outlined in the following two timetables, respectively. (**c**) IHC images on day 6 of LTL+/– cells cultured on 2D plates. The LTL+ fraction clearly

contains cells positive to LTL and megalin, where the apical expression of the latter is indicated by green arrowheads. There is no trace of LTL or megalin in the LTL– fraction. Scale bars, 100 μm. (**d**) Relative gene expression levels of four major proximal tubule markers corresponding to those of ACTB obtained from the cells, as sorted, (white frame) and after 7 days of culture in 2D plates (cyan frame), in logarithmic scale. "Organ. D22" refer to the organoid cells dissociated prior to storing on day 22. LTL+ and LTL– refer to the positive and negative fraction of the cells, as sorted, respectively. Tissue cultures of all the three groups were also examined (cult.). Expression levels of PT-specific genes decreased considerably upon culture in 2D conditions. Data belong to N = 1 independent experiment with a sample size (PCR replicates) of n = 3.

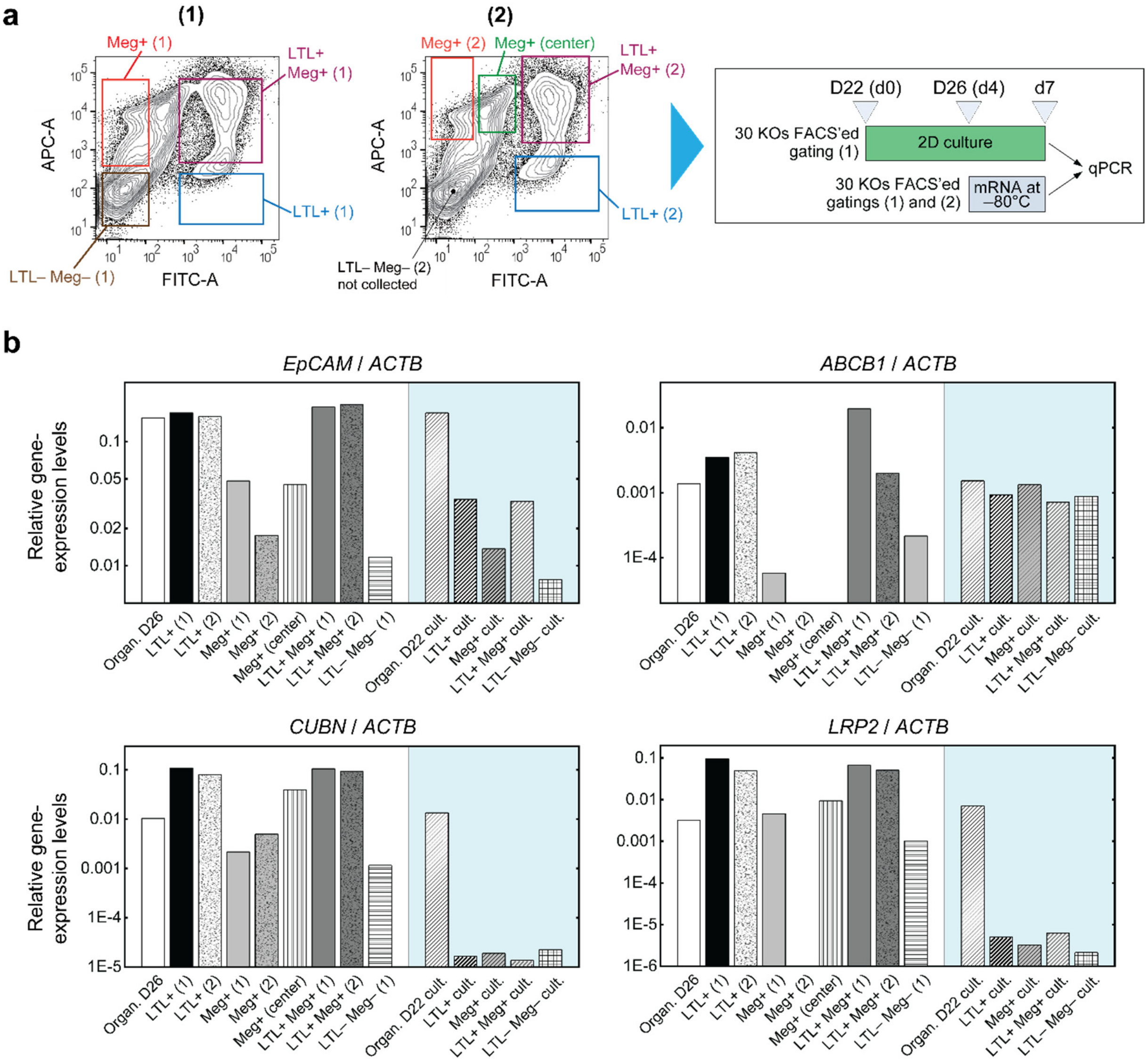

Supplementary Figure 2 | Attempts to characterize PT-like cells extracted from hiPSC-derived KOs through FACS using two markers (LTL and megalin). (**a**) Flow cytometry contour plots represent the gating strategies and densities of various cell populations for double-marker sorting schemes. Two attempts/gatings were made to sort the cells using LTL and megalin: (1) & (2). Alexa fluor 647 anti-rabbit (1/500) and Streptavidin FITC (1/500) were used as the secondary antibodies to tag megalin and LTL, respectively. The gating strategies for the cells to be sorted are shown. The timetable illustrates two experiments for mRNA preparation. In the first experiment 30 KOs were harvested and sorted through gating scheme (1) and then cultured for 7 days. In the second experiment 30 KOs were sorted through gating schemes (1) and (2) and them immediately lysed for mRNA extraction. (**b**) Relative gene expression levels of four major PT markers to those of ACTB obtained from the cells, as sorted, (white frame) and after 7 days of culture in 2D plates (cyan frame) via single-marker and double marker approaches, respectively. “Organ. D22” and “Organ. D26” refer to the organoid cells dissociated prior to storing on day 22 and 26, respectively. LTL+ (1&2) and Meg+ (1&2) refer to the cells positive to only LTL and only megalin, respectively, whereas LTL+ Meg+ (1&2) and LTL– Meg– (1) refer to the double positive and double

negative cells, as-sorted, respectively. Meg+ (center) embodies the small unknown population at the proximity of Meg+ (2). As indicated in the timetable of **a**, cells obtained from gating (1) were cultured (cult.). Expression levels of PT-specific genes decreased considerably upon culture in 2D conditions. LTL+ and double positive cells (LTL+ Meg+), as sorted, exhibited the highest levels of all the four genes examined. For LTL+ Meg+ cells, as sorted, there were no tangible differences between the 1st and 2nd gating attempts. Data belong to one independent experiment with a sample size (PCR replicates) of *n* = 3.

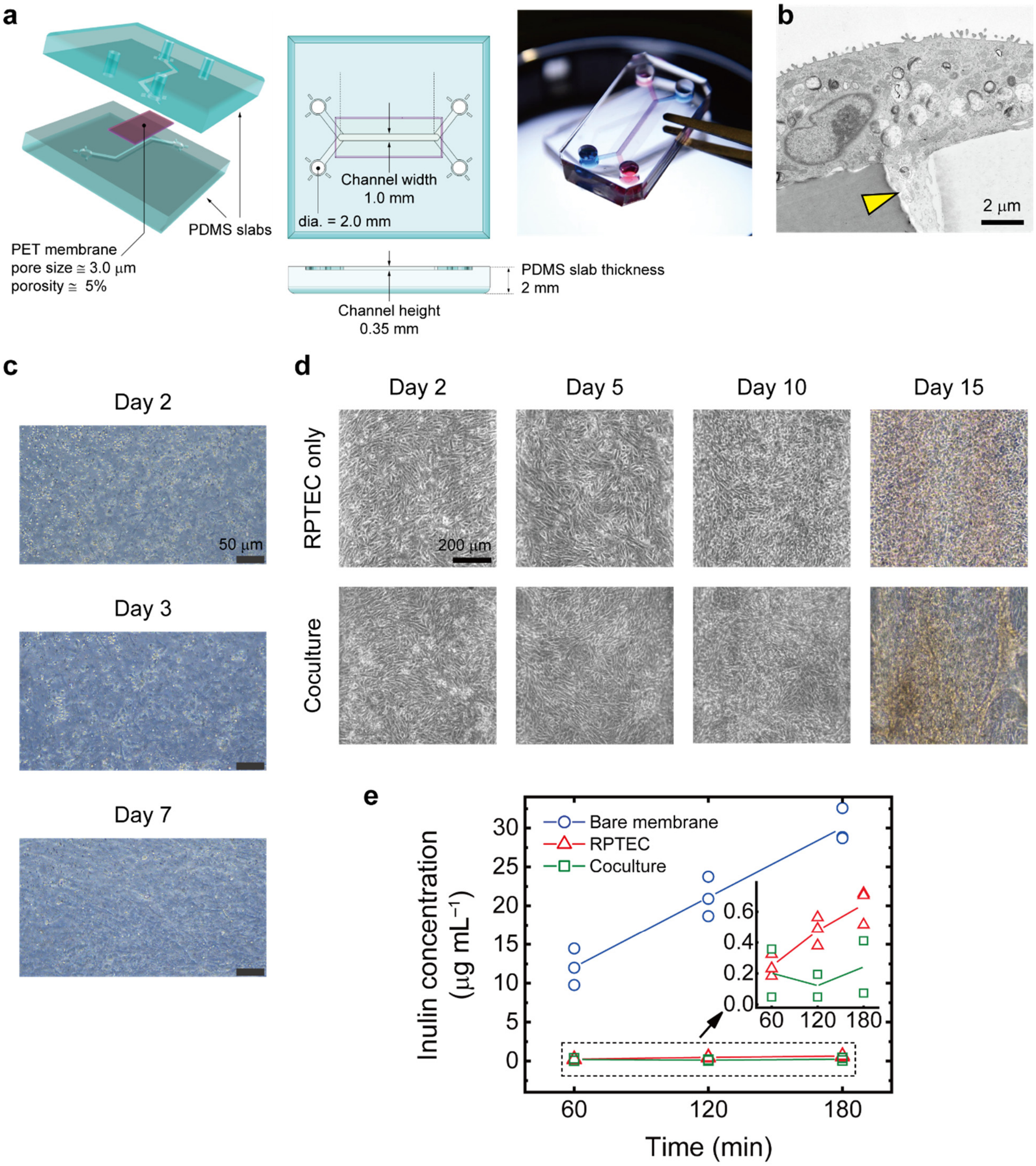

Supplementary Figure 3| Construction of the PToC. (**a**) A 3D schematic representation and a photograph of the device. Isolated top and bottom channels filled with blue and red inks, respectively, are discernable. (**b**) Representative TEM image showing penetration of the ECM components of an RPTEC into a 3 µm wide membrane pore (yellow arrowhead). Scale bar is 2 µm. (**c**) Bright field images showing the LTL– cells on the chip developing into a fibroblast like tissue and eventually disintegrating. Scale bar is 50 µm. (**d**) Bright field images showing the evolution of the 2D proximal tubule tissues formed on the PET membranes of the devices with ITO electrodes. Apart from the smaller cell size in the cocultured tissue, both layers are

morphologically similar up to day 15 where some aggregations began to appear in the coculture. Scale bar is 200 μm. (**e**) FITC-labeled inulin (3000 Da < MW < 6000 Da), applied at 100 μg mL^{-1} to the top channel (apical side) and measured at the bottom on day 14, is notably blocked by RPTEC and LTL+/RPTEC (coculture) cell-laden membranes. Impermeability of the tissue layers to inulin confirm the epithelial barrier function and validate active transport of albumin, glucose, and Rh123. N = 2 independent chips for the coculture and N = 3 independent chips for the bare membrane and RPTEC-only cases. Lines connect the average values.

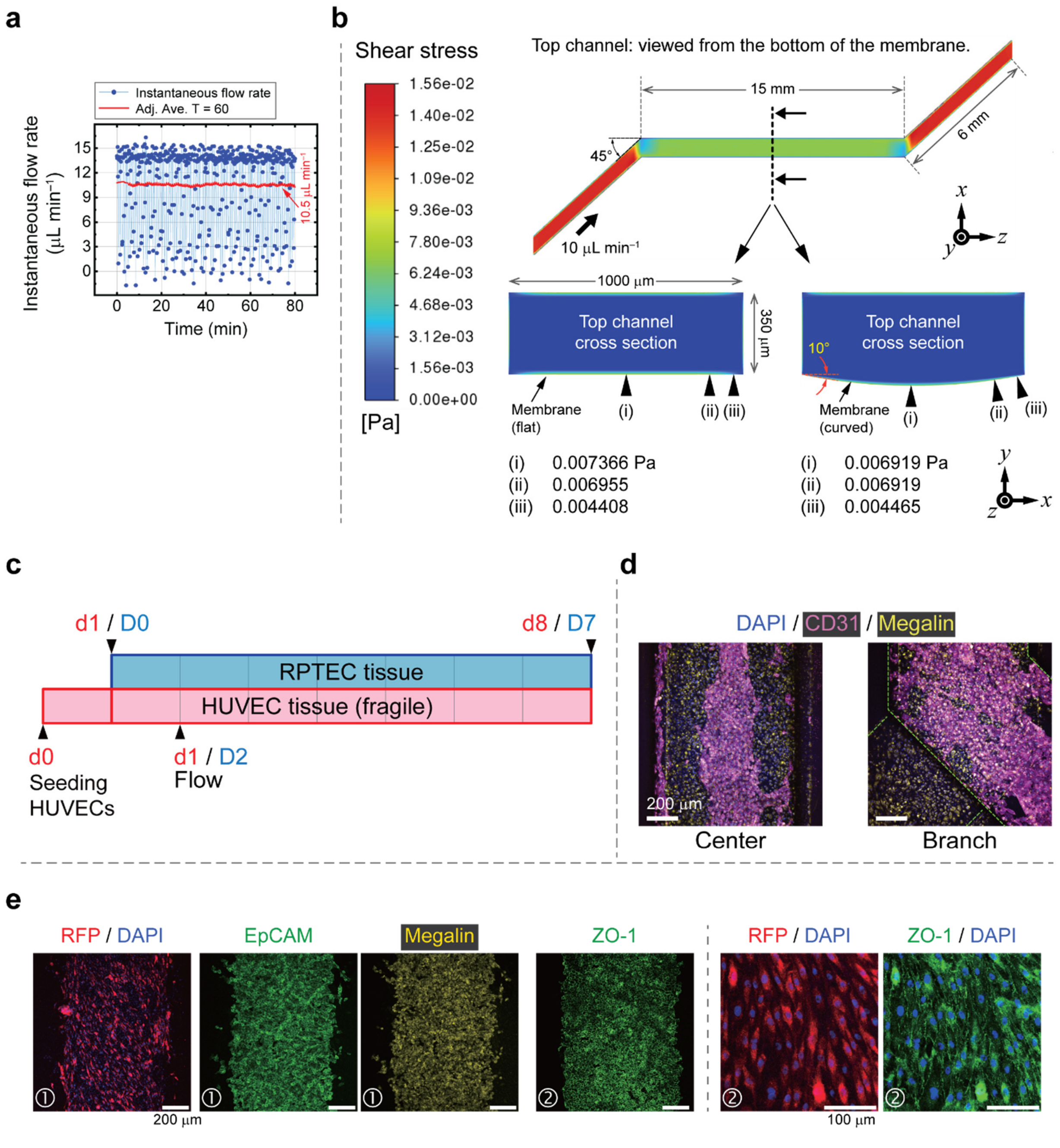
a
Instantaneous flow rate
Adj. Ave. T = 60
Instantaneous flow rate (µL min⁻¹)
10.5 µL min⁻¹
Time (min)
b
Shear stress
[Pa]
1.56e-02
1.40e-02
1.25e-02
1.09e-02
9.36e-03
7.80e-03
6.24e-03
4.68e-03
3.12e-03
1.56e-03
0.00e+00
Top channel: viewed from the bottom of the membrane.
15 mm
6 mm
45°
10 µL min⁻¹
1000 µm
350 µm
Top channel cross section
Membrane (flat)
(i) (ii) (iii)
Top channel cross section
10°
Membrane (curved)
(i) (ii) (iii)
(i) 0.007366 Pa
(ii) 0.006955
(iii) 0.004408
(i) 0.006919 Pa
(ii) 0.006919
(iii) 0.004465
c
d1 / D0
d8 / D7
RPTEC tissue
HUVEC tissue (fragile)
d0
Seeding HUVECs
d1 / D2
Flow
d
DAPI / CD31 / Megalin
200 µm
Center
Branch
e
RFP / DAPI
EpCAM
Megalin
ZO-1
RFP / DAPI
ZO-1 / DAPI
200 µm
100 µm

Supplementary Figure 4| Some details pertinent to the perfusion culture system and appearance of confluent tissue layers on the PToC. (**a**) Instantaneous rate of flow through one channel measured at Δt = 10 s intervals. The cyclical fluctuations are a signature of the rotary peristaltic pump. The red line indicates the adjacent average with a window of T = 60 points or 600 s. (**b**) Finite element modeling of the fluid flow in the upper channel to compute the amplitude of sheer stress on the membrane. The contour plots represent shear stress in Pa (1 Pa = 10 dyn cm^{-2}). Computed values agree well with the estimated value (0.06 dyn cm^{-2} using equation (1) in the 'methods' section of the text). It also appears that the curvature does not affect the values noticeably throughout the lion's share of the membrane area. (i) (ii) and (iii) indicate three sample points on the membrane where shear stress was probed. (**c**) Pilot seeding protocol incorporated with HUVECs being seeded first. (**d**) Z-intensity projected confocal fluorescent images taken from the central and branching regions of the bilayer microfluidic device, showing pronounced detachment of HUVECs in the former region. Since the microchannels completely overlap at the central region, there is higher chance that EGM2 becomes contaminated with REGM at this area. Such contamination is known to be the cause of HUVEC delamination (data not discussed herein). In the branching regions, at both ends, the probability of media intermix is less than that in the central part, therefore, the HUVECs remained attached until d8. Scale bars are 200 μm. (**e**) Confocal fluorescent images of immunostained RPTEC and RFP-tagged HUVEC tissue layers on D17/d7 obtained by the modified protocol signifying the fact that the entire membrane area is covered. RPTECs were identified by EpCAM and megalin. Notice the abundance and uniformity of tight junctions throughout the epithelial layer. ZO-1 was also expressed in RFP-HUVECs. Two distinct samples that were used for such demonstration (indicated by ① and ②). Scale bars are 200 μm on the leftmost four pictures and 100 μm on the rightmost two, respectively.

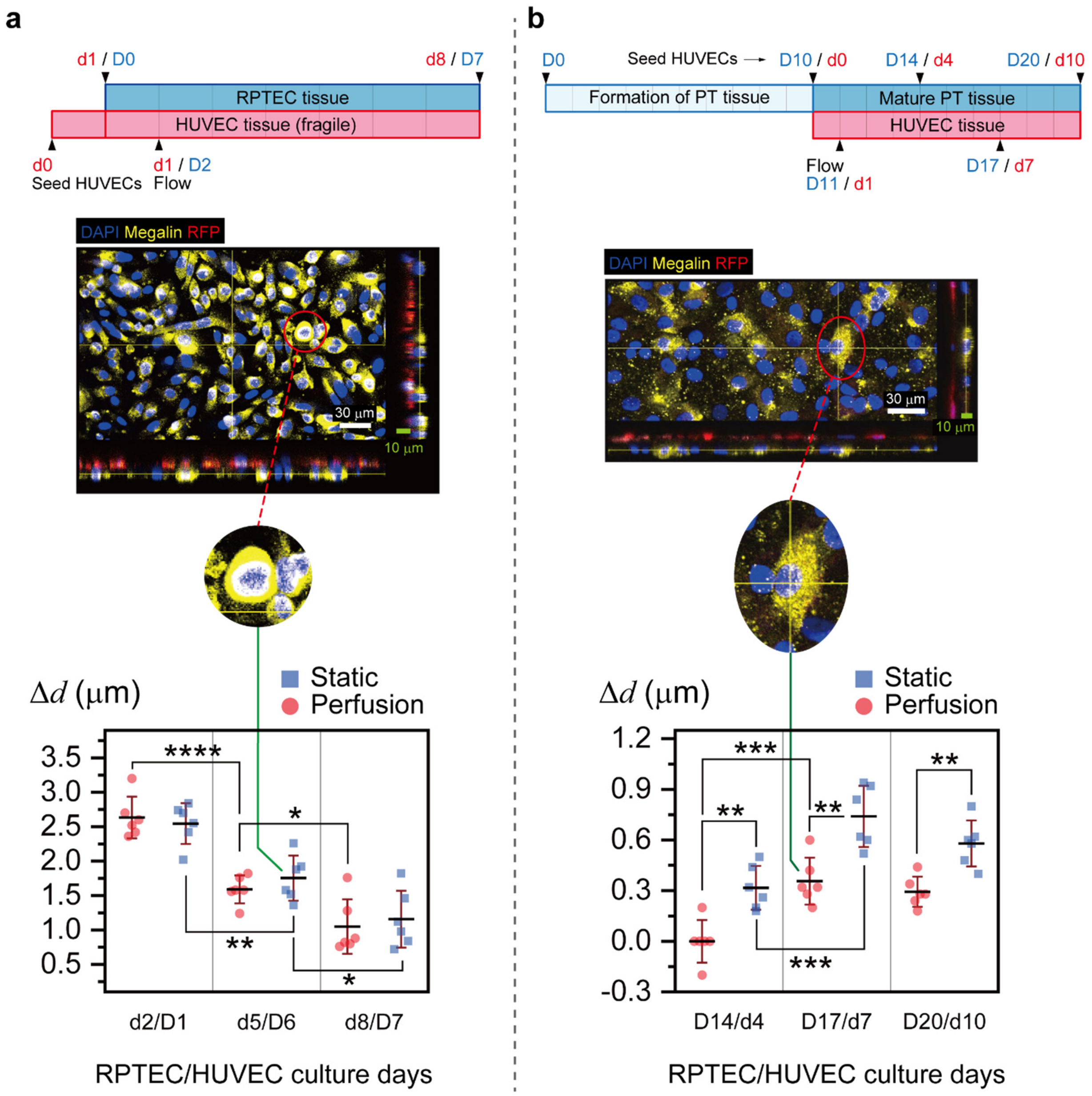

Supplementary Figure 5| Exemplifying how protein-to-nucleus distance is calculated from z-intensity profiles and presented in the plots of Fig. 5c-e in the main text. Representative fluorescent confocal images of the bilayer show the cases when (**a**) HUVECs or (**b**) RPTECs were seeded first. A single data point is obtained from each picture (e.g. for megalin). Magnified views around a single cell shows that megalin is condensed at the apical side in **a** whereas in **b**, it is spread in the cytosol. N = 2 independent chips were used to make n = 3 random measurements from each; Error bars indicate standard deviation. Statistically significant differences between data pairs are indicated by asterisks, *, **, ***, ****, for $p \leq 0.05$, 0.01, 0.001, and 0.0001, respectively.

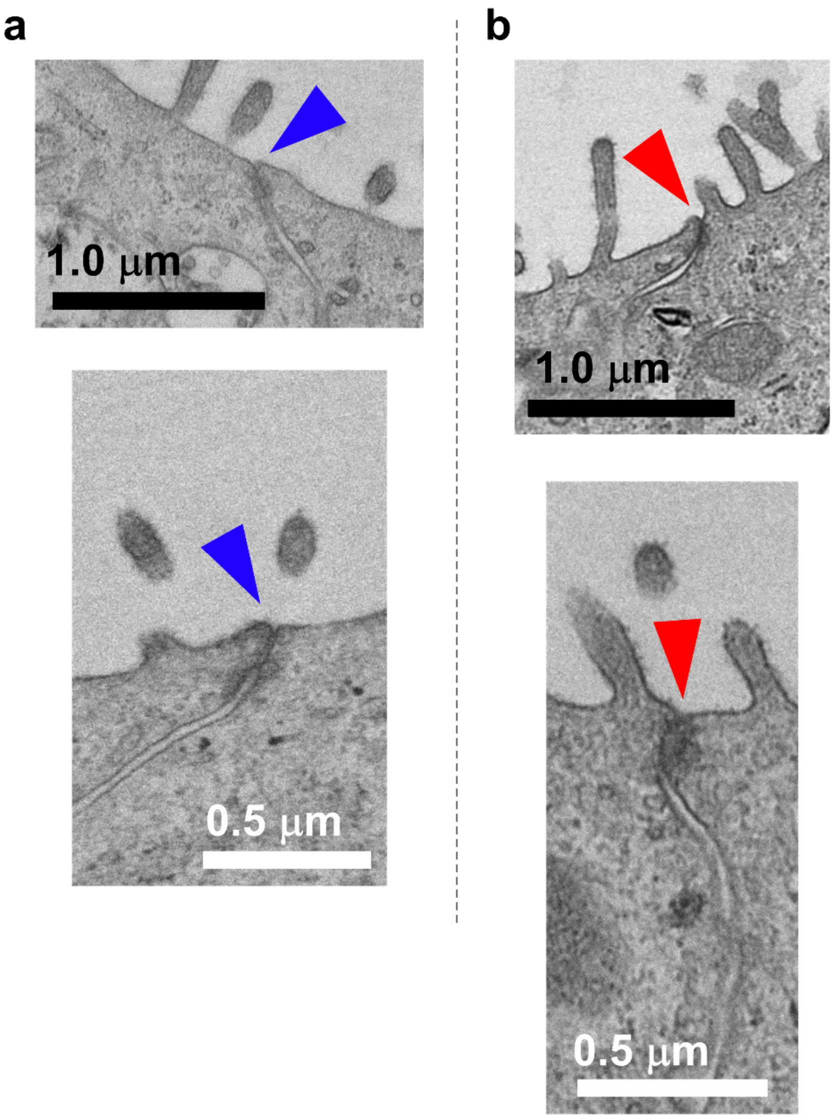

Supplementary Figure 6| High magnification TEM snapshots of RPTECs (day 14) focused on tight junctions (arrowheads) formed under (**a**) static and (**b**) perfusion culture conditions.

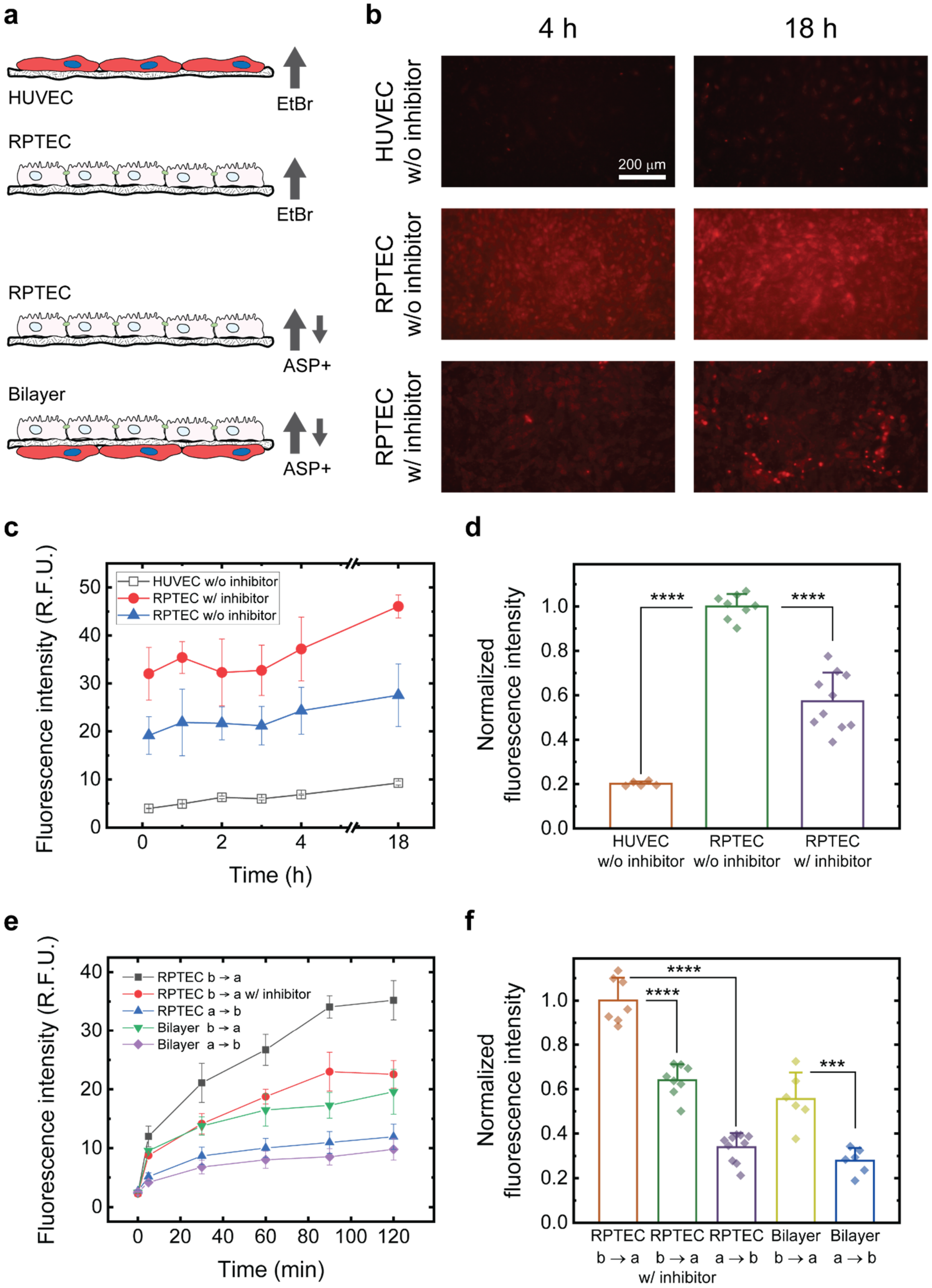
a
HUVEC
EtBr
RPTEC
EtBr
RPTEC
ASP+
Bilayer
ASP+
b
4 h
18 h
HUVEC w/o inhibitor
RPTEC w/o inhibitor
RPTEC w/ inhibitor
200 μm
c
Fluorescence intensity (R.F.U.)
Time (h)
HUVEC w/o inhibitor
RPTEC w/ inhibitor
RPTEC w/o inhibitor
d
Normalized fluorescence intensity
****
****
HUVEC w/o inhibitor
RPTEC w/o inhibitor
RPTEC w/ inhibitor
e
Fluorescence intensity (R.F.U.)
Time (min)
RPTEC b → a
RPTEC b → a w/ inhibitor
RPTEC a → b
Bilayer b → a
Bilayer a → b
f
Normalized fluorescence intensity
****
****
***
RPTEC b → a
RPTEC b → a w/ inhibitor
RPTEC a → b
Bilayer b → a
Bilayer a → b

Supplementary Figure 7| Demonstration of the OCT2 function using cationic fluorescent probes applied to the RPTEC single layer and RPTEC/HUVEC bilayer systems. (**a**) Schematic illustration of various configurations used to quantify uptake of EtBr and ASP+. (**b**) Fluorescent images of the tissue layers showing the uptake of EtBr introduced from the basal side. It is clear that the uptake is predominant in the RPTEC single layer and in the absence of the OCT2 inhibitor, cimetidine. HUVEC single layer tissues used as the control experiment showed no uptake of EtBr. Scale bar, 200 µm. (**c**) Time course evolution of EtBr uptake obtained by analyzing the fluorescence intensity of time lapse images. (**d**) Bar charts showing the average fluorescent intensity of samples obtained 18 hr after introduction of EtBr. The values were normalized to that of RPTEC single layer w/o inhibitor. Cimetidine reduced the uptake by half in RPTEC-only tissue layer. (**e**) Time course representation of ASP+ concentration up taken by RPTEC layers in two directions and in various conditions. (**f**) Fluorescent intensities of samples 120 min after exposure to ASP+ and normalized to that of the RPTEC single layer administered at b $\rightarrow$ a direction. All cases are w/o cimetidine, unless noted otherwise. N = 5 (minimum) measurements and error bars represent standard deviation of data.

Supplementary Table 2| 2NBDG uptake (a → b) rates in μg h^{-1} in various conditions and in the absence of SGLT2 inhibitor.

	RPTEC	Bilayer	Coculture
Static	0.36	0.50	0.59
Perfused	0.52	1.29	1.31

Supplementary Table 3| Rh123 efflux ratios, P_{app} (b→a) / P_{app} (a→b), in various conditions and in the absence of Pgp inhibitor.

	RPTEC	Bilayer	Coculture
Static	7.28	6.14	13.84
Perfused	15.25	14.02	16.68

Supplementary Table 4| List of antibodies used and their concentrations.

Antibody name	Supplier	Part no.	Concentration (dilution factor)
Anti-CD31	abcam	ab215912	1/300
Anti-CD326 (EpCAM)	Miltenyi Biotec	130-113-263	1/200
Anti-Collagen IV	abcam	ab6586	1/300
Anti-Laminin	abcam	ab11575	1/300
Anti-Lrp2 / Megalin	abcam	ab85626	1/300
Anti-ZO-1	ThermoFisher	ZO1-1A12	1/200
LTL Biotinylated	Vector Labs	B-1325-2	1/200
Phalloidin-iFluor 647	abcam	ab176759	1/1000

Supplementary Table 5| List of TaqMan primers used for qPCR analysis.

Gene	Alias	Assay ID
ACTB	ACTB	Hs01060665_g1
AQP1	AQP1	Hs01028916_m1
CDH1	Cadherin 1 (E-Cadherin)	Hs00170423_m1
CDH6	Cadherin 6 (K-Cadherin)	Hs00191832_m1
CUBN	Cubulin	Hs00153607_m1
EpCAM	EpCAM	Hs00901885_m1
ABCB1	MDR1 (Pgp)	Hs00184500_m1
LRP2	Megalin	Hs00189742_m1
SLC12A1	NKCC2	Hs00165731_m1
SLC22A6	OAT1	Hs00537914_m1
SLC22A2	OCT2	Hs01010723_m1
SLC3A1	RBAT	Hs00942976_m1
SLC5A2	SGLT2	Hs00894642_m1

Supplementary Table 6| BSA-AF488 diffusion rates compared between membranes with no cell and those with HUVEC only layers, in static and perfused cases.

	Diffusion rates (μg mL^{-1} h^{-1})	
	Bare membrane	HUVEC (day 4)
Static	0.115	0.113
Perfused	1.075	0.081

Supplementary Table 7| Calibration details for the fluorescent substrates used in this study. Gain refers to the slope of the standard curves mapping the fluorescent intensity onto their concentrations.

	Concentration range	Gain
BSA-AF488	1 – 10 μg mL^{-1}	1.46×10^3 RFU / (μg mL^{-1})
2NBDG	1 – 10 μM	1.10×10^3 RFU / μM
Rh123	0.05 – 1.25 μM	1.02×10^8 RFU / μM
FITC-labeled inulin (3000 Da < MW < 6000 Da)	0 – 50 μg mL^{-1}	6.17×10^6 RFU / (μg mL^{-1})
