# Supplementary material for: Cells sorted off hiPSC-derived kidney organoids coupled with immortalized cells reliably model the proximal tubule
Source: Commun Biol. 2023 May 4;6:483. doi: 10.1038/s42003-023-04862-7 (PMC10160057; doi:10.1038/s42003-023-04862-7)
Supplement: Supplementary file 2 — Description of Additional Supplementary Files [file 42003_2023_4862_MOESM2_ESM.docx]

**Description of Additional Supplementary Files**

**File name:** Supplementary Data 1

**Description:** Flow cytometry cell population data pertinent to Suppl. Figures 1 & 2

**File name:** Supplementary Movie 1

**Description:** Immunostained kidney organoid on day 22 against LTL (red). Cell nuclei are marked with DAPI (blue).

**File name:** Supplementary Movie 2

**Description:** Immunostained kidney organoid on day 22 against megalin (yellow). Cell nuclei are marked with DAPI (blue).

**File name:** Supplementary Movie 3

**Description:** Primary RPTECs marked with LTL (cyan), and their cell nuclei marked with YO-PRO-1 (green).

**File name:** Supplementary Movie 4

**Description:** LTL+ MACS’ed cells marked with LTL (cyan) and megalin (red), and their cell nuclei marked with YO-PRO-1 (green).

**File name:** Supplementary Movie 5

**Description:** LTL– MACS’ed cells immunostained with LTL (cyan) and megalin (red), and their cell nuclei marked with YO-PRO-1 (green).

**File name:** Supplementary Movie 6

**Description:** LTL+ FACS’ed cells marked with LTL (cyan) and megalin (red).

**File name:** Supplementary Movie 7

**Description:** Confocal laser scan from apical to basal sides of LTL+ cells cocultured with RPTEC/TERT1, focused on the aggregates, to show cell polarity in the coculture. Pgp (magenta) is the apical marker. Cell nuclei are marked with DAPI (cyan).

**File name:** Supplementary Movie 8

**Description:** Confocal laser scan from apical to basal sides of LTL+ cells cocultured with RPTEC/TERT1, focused on the aggregates, to show cell polarity in the coculture. LTL (blue) and ZO1 (red) are the apical markers. Cell nuclei are marked with DAPI (cyan).

**File name:** Supplementary Movie 9

**Description:** Confocal laser scan from apical to basal sides of LTL+ cells cocultured with RPTEC/TERT1, to show cell polarity in the coculture. LTL (red) and EpCAM (green) are the apical and basolateral markers, respectively. Cell nuclei are marked with DAPI (blue).

**File name:** Supplementary Movie 10

**Description:** Confocal fluorescent image of the bilayer scanned in z-direction. The RPTEC layer is revealed by SGLT2 (yellow) while RFP-HUVECs are revealed by CD31 (green). Cell nuclei are marked with DAPI (blue).

**File name:** Supplementary Movie 11

**Description:** 3D reconstructed image of the bilayer with RPTECs revealed by SGLT2 (yellow) and HUVECs revealed by CD31 (green).
