# Supplementary material for: Cells sorted off hiPSC-derived kidney organoids coupled with immortalized cells reliably model the proximal tubule
Source: Commun Biol. 2023 May 4;6:483. doi: 10.1038/s42003-023-04862-7 (PMC10160057; doi:10.1038/s42003-023-04862-7)
Supplement: Supplementary file 15 — Reporting Summary [file 42003_2023_4862_MOESM15_ESM.pdf]

nature portfolio

Corresponding author(s): Ryuji Yokokawa

Last updated by author(s): Apr 2, 2023

# Reporting Summary

Nature Portfolio wishes to improve the reproducibility of the work that we publish. This form provides structure for consistency and transparency in reporting. For further information on Nature Portfolio policies, see our Editorial Policies and the Editorial Policy Checklist.

## Statistics

For all statistical analyses, confirm that the following items are present in the figure legend, table legend, main text, or Methods section.

| n/a | Confirmed | |
|---|---|---|
| ☐ | ☒ | The exact sample size (*n*) for each experimental group/condition, given as a discrete number and unit of measurement |
| ☐ | ☒ | A statement on whether measurements were taken from distinct samples or whether the same sample was measured repeatedly |
| ☐ | ☒ | The statistical test(s) used AND whether they are one- or two-sided<br>*Only common tests should be described solely by name; describe more complex techniques in the Methods section.* |
| ☐ | ☒ | A description of all covariates tested |
| ☒ | ☐ | A description of any assumptions or corrections, such as tests of normality and adjustment for multiple comparisons |
| ☐ | ☒ | A full description of the statistical parameters including central tendency (e.g. means) or other basic estimates (e.g. regression coefficient) AND variation (e.g. standard deviation) or associated estimates of uncertainty (e.g. confidence intervals) |
| ☒ | ☐ | For null hypothesis testing, the test statistic (e.g. *F*, *t*, *r*) with confidence intervals, effect sizes, degrees of freedom and *P* value noted<br>*Give P values as exact values whenever suitable.* |
| ☒ | ☐ | For Bayesian analysis, information on the choice of priors and Markov chain Monte Carlo settings |
| ☒ | ☐ | For hierarchical and complex designs, identification of the appropriate level for tests and full reporting of outcomes |
| ☒ | ☐ | Estimates of effect sizes (e.g. Cohen's *d*, Pearson's *r*), indicating how they were calculated |

*Our web collection on statistics for biologists contains articles on many of the points above.*

## Software and code

Policy information about availability of computer code

| | |
|---|---|
| Data collection | Image J v1.53f51 from NIH; QuantStudio Design & Analysis v1.4.3; BD FACS Diva 8.0.2; Olympus FV31S-SW Version 2.4.1.198 ( Powered by H-PF Version 2.7.2.435 ) |
| Data analysis | OriginPro 2018 (64-bit) SR1 b9.5.1.195; Microsoft Excel 2016 MSO (16.0.4432.1000) 64-bit; FlowJo 10.5.3; Olympus FV31S-SW Version: 2.3.1.163 |

For manuscripts utilizing custom algorithms or software that are central to the research but not yet described in published literature, software must be made available to editors and reviewers. We strongly encourage code deposition in a community repository (e.g. GitHub). See the Nature Portfolio guidelines for submitting code & software for further information.

## Data

Policy information about availability of data

All manuscripts must include a data availability statement. This statement should provide the following information, where applicable:

- Accession codes, unique identifiers, or web links for publicly available datasets
- A description of any restrictions on data availability
- For clinical datasets or third party data, please ensure that the statement adheres to our policy

All data generated or analysed during this study are included in this published article and its supplementary information files. If any realavant source/raw data for figures/charts are required it can be provided by the corresponding author.

# Human research participants

Policy information about studies involving human research participants and Sex and Gender in Research.

| | |
|---|---|
| Reporting on sex and gender | Sex and gender were not considered in this study. Sex- and gender-based analyses were not performed. |
| Population characteristics | See above. |
| Recruitment | N/A |
| Ethics oversight | N/A |

Note that full information on the approval of the study protocol must also be provided in the manuscript.

# Field-specific reporting

Please select the one below that is the best fit for your research. If you are not sure, read the appropriate sections before making your selection.

☒ Life sciences ☐ Behavioural & social sciences ☐ Ecological, evolutionary & environmental sciences

For a reference copy of the document with all sections, see nature.com/documents/nr-reporting-summary-flat.pdf

# Life sciences study design

All studies must disclose on these points even when the disclosure is negative.

| | |
|---|---|
| Sample size | Different criteria were used to determine/set the sample size for each experiment. To collect transport data from MPS chips a minimum sample size of n = 3 chip was used. For qPCR analysis reported in the main text n = 5 wells (of a 96 well plate) was used for each gene/sample and the experiments were repeated N = 3 times, that is 3 independent trials. For qPCR data reported in Supplementary Figures 1d and 2b while n = 5 wells were used, there was only one trial. |
| Data exclusions | Data were excluded from cell-laden microphysiological system (MPS) chips provided they had displayed transport rates significantly higher than the average. Briefly, surfaces of cell-laden PET membranes were examined under optical microscope immediately after the transport tests to ensure there are entirely covered with the confluent tissue and no pin holes exist. Evidently, devices with pin holes had displayed transport rates significantly higher than the rest. A total of minimum n = 3 leak free devices were used. |
| Replication | In particular: For qPCR experiments, three independent experiments for each condition (e.g. from initial cell seeding, culture, perfusion flow, etc. were carried out and there were all reproducible.<br>For transport measurements, we used 3 non-leaking devices as outlined above.<br>For the FACS/qPCR data presented in Supp. Fig. 1d & 2b, only one experiments was carried out, as a result the reproducibility cannot be 100% confirmed. |
| Randomization | Allocations were random, e.g. MPS chips were enumerated 1,2,3, ... in order to facilitate tracing the acquired data back to the chip. |
| Blinding | All the investigators were blinded to group allocations both during data collection and analysis. |

# Reporting for specific materials, systems and methods

We require information from authors about some types of materials, experimental systems and methods used in many studies. Here, indicate whether each material, system or method listed is relevant to your study. If you are not sure if a list item applies to your research, read the appropriate section before selecting a response.

## Materials & experimental systems

| n/a | Involved in the study | |
|---|---|---|
| ☐ | ☒ | Antibodies |
| ☐ | ☒ | Eukaryotic cell lines |
| ☒ | ☐ | Palaeontology and archaeology |
| ☒ | ☐ | Animals and other organisms |
| ☒ | ☐ | Clinical data |
| ☒ | ☐ | Dual use research of concern |

## Methods

| n/a | Involved in the study | |
|---|---|---|
| ☒ | ☐ | ChIP-seq |
| ☐ | ☒ | Flow cytometry |
| ☒ | ☐ | MRI-based neuroimaging |

## Antibodies

| | |
|---|---|
| Antibodies used | In alphabetical order:<br><br>1. Primary antibodies:<br>Anti-CD31 ab215912 abcam;  Anti-CD326 (EpCAM) 130-113-263 Miltenyi Biotec;  Anti-Collagen IV ab6586 abcam;  Anti-Laminin ab11575 abcam;  Anti-Lrp2 / Megalin ab76969 abcam;  Anti-P Glycoprotein ab170904 abcam;  Anti-SGLT2 ab85626 abcam;  Anti-ZO-1 ZO1-1A12 ThermoFisher;  Lotus Tetragonolobus Lectin (LTL) Biotinylated B-1325-2 Vector Labs;  Phalloidin-iFluor 647 for F-actin ab176759 abcam<br><br>2. Secondary antibodies and nucleic acid stains:<br>Alexa Fluor® 647 Anti-Biotin 200-602-211 Jackson ImmunoResearch;  Goat Anti-Mouse IgG H&L (Alexa Fluor® 488) ab150113 abcam;  Goat Anti-Rabbit IgG H&L (Alexa Fluor® 568) ab175471 abcam;  Streptavidin, Alexa Fluor™ 405 conjugate S32351 ThermoFisher;  YO-PRO™-1 Iodide (491/509) Y3603 ThermoFisher;  DAPI D3571 ThermoFisher |
| Validation | For proximal tubule-specific antibodies, HUVECs were used as the negative control. |

## Eukaryotic cell lines

Policy information about cell lines and Sex and Gender in Research

| | |
|---|---|
| Cell line source(s) | 1. RPTEC/TERT1: hTERT-immortalized epithelial cell, isolated from the proximal tubule of a male patient: Obtained from ATCC (CRL-4031™)<br><br>2. hiPSCs: female CRL1502-C32 fibroblasts derived from ATCC CRL-1502 fetal fibroblasts, as outlined in:<br>J. A. Briggs et al., Stem Cells, vol. 31, no. 3, pp. 467-478, 2013.<br><br>3. H2B-GFP hiPSC line, was derived from 1502.3 hiPSC line and has H2B-GFP driven by CAG promoter in the AAVS1 locus. |
| Authentication | None of the cell lines used were authenticated. |
| Mycoplasma contamination | All cell lines were tested negative for mycoplasma contamination. |
| Commonly misidentified lines<br>(See ICLAC register) | N/A |

## Flow Cytometry

### Plots

Confirm that:

☒ The axis labels state the marker and fluorochrome used (e.g. CD4-FITC).

☒ The axis scales are clearly visible. Include numbers along axes only for bottom left plot of group (a 'group' is an analysis of identical markers).

☒ All plots are contour plots with outliers or pseudocolor plots.

☒ A numerical value for number of cells or percentage (with statistics) is provided.

### Methodology

| | |
|---|---|
| Sample preparation | Please see item #6 (FACS) of the METHODS section in the main text. |
| Instrument | BD FACSAria™ III |
| Software | BD FACS Diva 8.0.2 was used to acquire data. FlowJo 10.5.3 was used to analyze and plot flow cytometry data. |
| Cell population abundance | We have quantified relevant cell populations. Please refer to the attached excel file. |
| Gating strategy | Please see Supplementary Figures 1 and 2 (updated). |

☒ Tick this box to confirm that a figure exemplifying the gating strategy is provided in the Supplementary Information.
